# Marine Gastrotricha of the Near East: 1. Fourteen new species of Macrodasyida and a redescription of *Dactylopodola agadasys* Hochberg, 2003

**DOI:** 10.3897/zookeys.94.794

**Published:** 2011-05-03

**Authors:** William D. Hummon

**Affiliations:** Department of Biological Sciences, Ohio University, Athens, Ohio 45701 USA

**Keywords:** Cyprus, Egypt, Israel, Australia, Mediterranean Sea, Red Sea, Meiofauna, Systematics

## Abstract

The near eastern geographical region is almost devoid of reports of macrodasyidan gastrotrichs, the exceptions themselves being part of this study. Here, as Part 1 are described fourteen new Macrodasyida from countries of the Near East (Cyprus, Egypt and Israel, representing both the Mediterranean and the Red Seas), and a redescription of the previously described Dactylopodolidae: *Dactylopodola agadasys* Hochberg, 2002. The new species are: Cephalodasyidae (2) - *Cephalodasys dolichosomus*; *Cephalodasys saegailus*; Dactylopodolidae (1) *Dendrodasys rubomarinus*; Macrodasyidae (5) - *Macrodasys imbricatus*; *Macrodasys macrurus*; *Macrodasys nigrocellus*; *Macrodasys scleracrus*; *Urodasys toxostylus*; Thaumastodermatidae(4) - *Tetranchyroderma corallium*; * Tetranchyroderma rhopalotum*; *Tetranchyroderma sinaiensis*; *Tetranchyroderma xenodactylum*; Turbanellidae(2) - *Paraturbanella levantia*; *Turbanella erythrothalassia* - **spp. n.**

## Introduction

As of December 2010 there are 305 described species of Macrodasyida, of which 142 species belong in the family Thaumastodermatidae, 56 in the family Turbanellidae and 45 in the family Macrodasyidae, the three most diverse families, and 72 species in the genus *Tetranchyroderma*, 32 in the genus *Macrodasys* and 28 in the genus *Turbanella*, the three most diverse genera in the order. Most of the species contained in this report also belong to the above taxa; they come from two oceanic regions, the eastern Mediterranean Sea and the northern Red Sea. These were part of the fauna that were encountered in Egypt and Cyprus during an eight month Fulbright Research Fellowship from April through November 1994 with the Department of Oceanography in the University of Alexandria. Three trips were also made to Israel, one in February 1992, a second in February 1999, and a third in September 1999, the last covering a full month’s time based at the Department of Zoology at Hebrew University, Jerusalem. This is the first of several such manuscripts, this one on the Macrodasyida, the second forthcoming will be on the known species of Gastrotricha in the region, and a third on new members of the order Chaetonotida.

Basically, work in this geographical region began with Masry (1970) and Hulings (1971), surveying on physical-chemical parameters and meiofauna of the Mediterranean Sea, namely Lebanon and Israel in the Near East. Both found Gastrotricha, but recorded none to species; they were concentrating on micro-crustaceans, and, of course, hardly any of the gastrotrich species that they might have seen had been described at that time. Progress reports on the gastrotrichs of the present study appeared as abstracts under Hummon and co-authors in the American Zoologist in 1992 (Hummon and Hummon 1992), ‘94 (Hummon et al. 1996) and ‘95 (Hummon and Hummon 1995). There exist several written reports of individual chaetonotidan species from this region [*Chaetonotus luporinii* in Balsamo et al. (1996); *Heteroxenotrichula pygmaea* in Hummon (2010a) and *Aspidiophorus paramediterranea* in Hummon (2010b)], whereas the reports of macrodasyidan species have come mostly as spinoff publications of this study, namely *Mesodasys adenotubulatus* in Hummon et al. (1993); *Turbanella bocqueti* in Hummon (2008); *Tetranchyroderma papii* in Hummon (2009); *Diplodasys sanctimariae* in Hummon and Todaro (2009); and *Dactylopodola typhle* in Hummon (2010a). Data from this study have been available for co-workers on CD (2001), more recently revised with the addition of additional described species and video sequences of described species, all being transferred to the server of Hummon (2004, 2007, 2009), with many of the collected data from the Mediterranean being referred to in a general way by Todaro at al. (2003) and with many videos being enumerated by Hummon et al. (2005). Of the new species being described here, two are Mediterranean, 12 Red Sea and but one species occurring in both.

## Methods and materials

**Figure 1.**
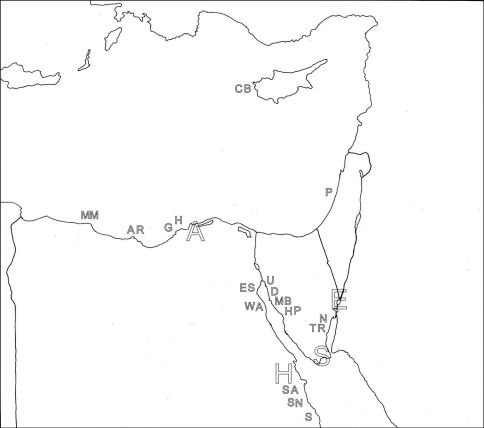
Map, with the collection locations listed in Table 1 indicated

Littoral collections were made by whole-beach transects, with 8–10 sites spaced more or less equally from lowest to highest water levels. Locations are presented in a table (Table 1) and a map (Figure 1); more detailed maps will accompany upcoming parts of the study. Sites were sampled at depth using a core tube (for wet samples) or shovel and plastic scoop (for dry samples). Sampling in the sand was mostly continuous, ranging from 0–10 cm (low water levels) to 0–30 (high water levels), usually reaching ground water levels. Sublittoral collections were made by scoop sampler, taken while wading, snorkeling or occasionally diving by SCUBA. Sand was placed in whirl-pak bags, kept at relatively cool temperatures and returned to the laboratory as soon as possible for analysis. In the lab, samples were keep with little loss of living material for up to 10 days, analysis being carried out as quickly as possible, beginning with earliest collected samples or samples with heavy organic loads.

**Table 1.** Locations in the Mid East that are referred to in the text, along with the alphabetic symbols that are used in Figure 1.

MEDITERRANEAN SEA			
	CYPRUS	CB – Coral Bay	
	EGYPT	MM - Marsa Matruh	
		AR - Sidi Abd al-Rahman	
		G - Green Beach	
		H - Hannoville	
		A - Alexandria {Betash ’Agami, Bir Mesud, Cleopatra, Mamura}	
	ISRAEL	P - Palmachim N	
RED SEA			
	EGYPT	ES - Ein Sukhna	
			16 km south of Ein Sukhna,
			23km south of Ein Sukhna,
		WA - Wadi ’Araba	
		U - ’Uyun Musa	
			Ras Sudr
		D - Daghashland	
		MB - Moon Beach	
		HP - Hammam Pharoan	
		H - Hurghada {Moon Valley, Giftun Village, Princess Village, Mugawish}	
		SA - Sharm el-Arab	
		SN - Sharm el-Naga	
		S - Safaga	
		G - Giftun Island S	
		AR - Abu Ramada 1, 2, 3	
		S - Sharm el-Sheikh {Ras Mohamed National Park: Main Beach, Marsa Bareika, Tip RM, West Gate}, {Far Garden, Middle Garden, Na’ama Bay, Nabq, Ras Nasrani, Ras Qanti}	
		TR - Tareef el-Reeh	
		N - Nuweiba	
	ISRAEL	E - Eilat {Princess Hotel, Snuba Dive Shop, North Beach, Coral Beach: S, M2, M3, M4, M5, N1}	

Analytic methods are more fully given in Hummon (2008). About 20 ml of sand was placed in a 125 ml plastic cup, 6 to 7 % MgCl2 added to cover, and after swirling the cup was left for 10 minutes. An equal amount of ambient sea-water was added, the contents swirled once again, and the supernatant poured into a 60 ml plastic Petri dish, followed by a similar amount of sea-water and another decantation to fill the Petri dish, its contents then being split between that and another Petri dish. After settling, the dish was inspected for now-revived gastrotrichs using a Wild M8 stereo-microscope at 18 to 20 × magnification. A desired specimen was transferred to a drop of MgCl2 on a microscope slide, oriented, covered with a 16 µm square coverslip with posts of non-toxic modeling clay at its corners. After marking the specimen’s location with a fine felt tip marker, the slide was transferred to a Nikon LabPhot II compound microscope with a differential interference contrast (DIC) optics, having numerical apertures (n.a.) for oil immersion objective lenses of 1.00 (for a 100× lens) and 1.25 (for a 63× lens). Videotaping with a Hi8 camcorder began at the highest magnification that would show the entire specimen (4 × objective for very long animals to 40 × for short animals), then again at higher magnifications. Videos have been digitized in wmv format, edited, and rendered into MPEG 2 (and MPEG 1) versions, and placed on a server for read-only downloading by other workers (Hummon et al. 2005; Hummon 2009).

Drawings of holotype specimens were made using a drawing tube on the compound microscope. My finished drawings involve both dorsal and ventral views as mirror images of one another, occasionally with a composite dorsal/ventral view of juveniles and/or subadults, and always with drawings of cuticular armature, when appropriate, all with appropriate scale bars. Several of the species described herein are based on subadults, which already show characters of the adults that distinguish them from other species. In addition to the species name, for each is included a unique ‘DOS name’ based on a three-letter designation for genus and a four-letter designation for the specific epithet, giving the eight-letter ‘title’ that originated when the MS-DOS (Microsoft Disk Operating System) would only allow eight letters for a file-name.

Morphological symbols and conventions are as follows: Lt: Length, total: from anterior tip of head to posterior tip of caudum and its adhesive tubes; LPh: Length, pharynx from anterior tip of head to PhJIn; PhJIn: Junction between pharynx and intestine; WHd /WNk /WTr /WFrBs /WFrTp: Width of head, neck, trunk, furcal base and furcal tips; TbA /TbL /TbD /TbV /TbP /TbS: adhesive tubes of the anterior, lateral, dorsal, ventral, posterior (caudal), and “Seitenfüsschen” series; U: Percentage units of Lt from anterior to posterior × 100; the ‘Adult’ condition is attained when evidence of reproductive maturity is attained: testes, ovules or accessory sex organs. Columns: longitudinal in orientation; Rows: transverse in orientation; the caret ^ refers to a type locality. Latitude and longitude are given for type localities (Ordinance Survey references are given for Israeli locations); those for all other localities can be found in the Global Data Base (Hummon 2009). Sea surface temperatures in the eastern Mediterranean range from 16°C in March to 26°C in August-September; those for the northern Red Sea are about 3°C higher throughout the year. Sea surface salinities in ppt (practical salinity units) for the two regions are not as variable from season to season and range in the upper 30’s in the eastern Mediterranean and in the lower 40’s in the northern Red Sea.

## Systematics

**Order MACRODASYIDA Remane, 1929 (Rao & Clausen, 1970)**

### Family CEPHALODASYIDAE Hummon & Todaro, 2010 Genus Cephalodasys Remane, 1926

#### Cephalodasys dolichosomus sp. n.

urn:lsid:zoobank.org:act:07C24BD1-E79E-4A7A-AC89-C64D37E718AE

http://species-id.net/wiki/Cephalodasys_dolichosomus

[Cfd dlsm]

Figure 2

Cephalodasys  EgyB Hummon (2009) [E Med & Red Sea Database].

##### Diagnosis:

 Adult Lt to 772 µm; PhJIn at U28. Head small, pyriform, with a broad circumcephalic band of cilia at U02–U05, separated from the rest of the body by a long gradual neck constriction; trunk narrow, bowed outward along the mid-gut, caudum slightly flaired. Epidermis finely granular, without glands. TbA 4 per side, borne on fleshy hands; TbVL 6 per side, with 5 inserted regularly from U27 to U51 and the 6th at U72, one in the rear pharyngeal region, the others in the fore- and midgut region; TbP 10–12, inserting on the rounded caudum. Locomotor ciliature: 2 longitudinal bands, separate from one another, but uniting with cilia of the transverse cephalic band in front, and joining together behind the anus. Mouth terminal, of medium diameter; buccal cavity cylindrical; pharyngeal pores basal; intestine broadest at the rear mid-gut, narrowing behind; anus ventral at U95. Hermaphroditic; testes begin at the PhJIn, vasa deferentia run rearward, but termini not seen; a column of eggs lies along the mid-gut, developing front to rear; frontal and caudal organs not seen.

##### Description:

**Figure 2.**
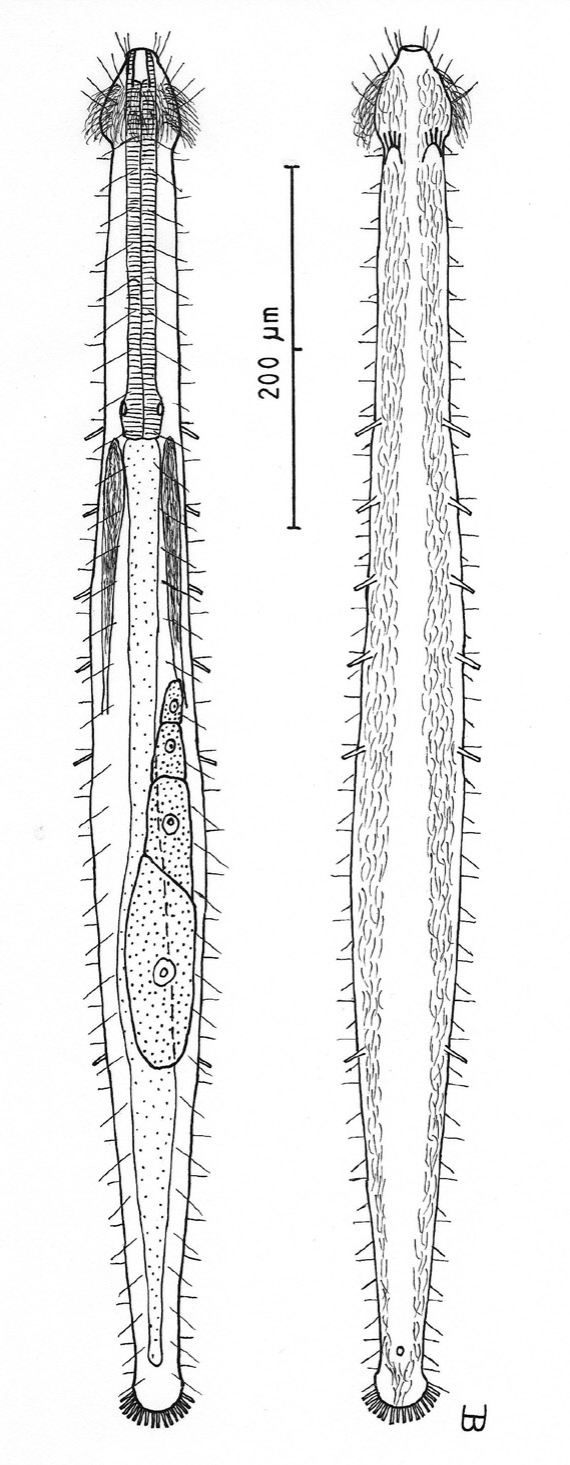
*Cephalodasys dolichosomus* sp. n. dorsal and ventral views of a mature adult (Lt=772, LPh=215 µm) from Main Beach, Ras Mohamed National Park, S. Sinai, Egypt; dorsal with dorsal and lateral body cilia, digestive and reproductive tracts; ventral with adhesive tubes and locomotor ciliary bands.

 Adult Lt 615–772 µm; LPh 205–215 µm to PhJIn at U33–U28 (Fig. 2). Body transparent, strap-shaped, dorsoventrally flattened, but vaulted dorsally; head small, pyriform, truncated apically, with a broad circumcephalic band of cilia at U02–U05, separated from the rest of the body by a long gradual neck constriction; trunk narrow, bowed slightly outward along the mid-gut, and then narrowing again to a rounded, slightly flaired caudum. Widths of head /neck /trunk /caudal base /caudal flair, and locations along the length of the body are as follows: 44 /32 /63 /24 /28 µm at U05 /U07 /U54 /U95 /U96, respectively. Cuticle is flexible, epidermis finely granular, but lacking glands.

*Adhesive tubes:*TbA 4 per side (L 8–9 µm), borne on broad fleshy hands that insert at U08; TbVL 6 per side (L 15–18 µm), 5 inserted regularly from U27 to U51 and the 6th at U72, one between pharyngeal pores and PhJIn, the others in the fore- and midgut region; TbP 16 total (L 7–10 µm), inserting in a single row about the flaired caudum.

*Ciliation:*A number of cilia insert on each side of the head (L 9–18 µm); a broad transverse band of cephalic cilia (L 18–20 µm) covers the broadest part of the head from U02 to U05 and joins the locomotor ciliature ventrally; some 30–32 sensory hairs occur in lateral (L 9–12 µm) and dorsolateral (L 18–20 µm) columns along either side of the trunk at U06–U96. Ventral locomotor ciliature forms two longitudinal bands of short (L 9–12 µm), scattered cilia that follow the lateral body contours beneath the entire length of the body, separate medially beneath the head and pharynx, but converging behind the anus and merging into a single band onto the caudum.

*Digestive tract:* Mouth is terminal, of medium width (6 µm diameter); buccal cavity nearly cylindrical, lined with cuticule of medium thickness; pharynx narrows toward the rear, but with basal pharyngeal pores showing dorsolateraly; intestine broadest in the rear mid-gut region, narrowing behind; anus is ventral at U95.

*Reproductive tract:* Hermaphroditic; testes begin at the PhJIn, vasa differentia lead rearward past U50, but termini not seen; ovary solitary, lying above the foregut on the right side, with a column of multiple ova developing toward the rear, reaching 115 × 40 µm in size; frontal and caudal organs not seen.

*Ecology:* Occasional in frequency of occurrence (10–30% of samples), scarce to prevalent in abundance (3% to more than 30% of a sample, the latter often a sub- **[sdom]** or co-dominant **[cdom]**); *littoral* in fine, well sorted sand to very fine-very coarse, poorly-extremely poorly sorted, clean to detrital sand at 0–10 cm depth, mean low water to extreme low water; mostly *sublittoral* in very fine to medium, well to medium sorted, clean silicious or coralline sands, sometimes mixed with shell and/or coral gravel, at 0.5–8 m water depth.

##### Geographical distribution:

 **Red Sea:** *EGYPT*: {23km south of Ein Sukhna, Marsa Bareika N **[sdom]**, ^Main Beach Ras Mohamed NP **[cdom]** (27°44'N, 34°12'E) [4-videos], Na’ama Bay N **[sdom]**, Sharm el-Sheikh [3-videos], Ras Sudr **[sdom]}**; *ISRAEL*: {Coral Beach M5 [2-videos]}.

##### Remarks:

 There are nine video sequences of *Cephalodasys dolichosomus* sp. n., all from the upper Red Sea in Egypt and Israel. Three of these are available as MPEG 2 (and MPEG 1) from Hummon (2009): #744 a mature adult **Lectotype** of Lt=772 µm (LPh=178 µm), collected in July 1994 from Main Beach, Ras Mohamed NP, S. Sinai, Egypt; #1917 a mature adult of Lt=763 µm (LPh=172 µm) from Coral Reserve, Eilat, Israel; and the other, #742 a subadult of Lt=186 µm (LPh=93 µm) from Sharm el-Sheikh, S. Sinai, Egypt. *Cephalodasys dolichosomus* can occupy a variety of sediments, but its greatest numbers occur sublittorally in finer, well sorted sands; it is unusual among the genus in having so many of its findings as co- or sub-dominant abundances.

##### Etymology:

 Dolichosoma (Greek: *dolichos + soma* = meaning ‘long body’) refers to the overall length of the animal, enhanced by the relative slenderness of its silhouette.

##### Taxonomic affinities:

When mature, *Cephalodasys dolichosomus* sp. n. is the longest, thinnest member of the genus, the only species in the genus that has the following combination of characters: a small pyriform head, with a transverse band of cephalic cilia, a PhJIn at U28, which also has TbA 4 per side; TbVL 6 per side (5 at U27–U51 and a 6th at U72); and TbP 16 total, borne on a flaired caudum; TbL *per se* /D/V absent; and ova developing fore to aft, though accessory sex organs were not seen. *Cephalodasys dolichosomus* belongs in the group of species that have pyriform heads, but is longer, thinner and with fewer TbL than other species with which it might be compared, namely *Cephalodasys cambriensis* (Boaden, 1963); *Cephalodasys littoralis* Renaud-Debyser, 1964; *Cephalodasys pacificus* Schmidt, 1974; and *Cephalodasys turbanelloides* (Boaden, 1960).

#### Cephalodasys saegailus sp. n.

urn:lsid:zoobank.org:act:61C1A2D5-F8EA-441A-AAB3-44F8C8B4883D

http://species-id.net/wiki/Cephalodasys_saegailus

[Cfd sagl]

Figure 3

Cephalodasys  EgyA of Hummon (2009) [E Med & Red Sea Database]; Todaro et al. (2003: Appx. 1).

##### Diagnosis:

 Adult Lt to 517 µm (perhaps reaching 600 µm); PhJIn at U38. Head pyriform, with a broad circumcephalic band of cilia at U02–U07, separated from the rest of the body by a long gradual neck constriction; trunk medium, broadest at the mid-body, caudum slightly flaired. Epidermis finely granular, without glands. TbA 4 per side, borne on fleshy hands; TbVL 10 per side, inserted regularly from U24 to U66, three along the rear pharynx, the others along the fore- and midgut; TbV 2 per side amid the TbVL series at U44 and U49; TbP 18 of varying lengths (12 longer, 6 shorter), inserting on the rear of the rounded caudum. Locomotor ciliature: 2 longitudinal bands, separate from one another, but uniting with cilia of the transverse cephalic band in front and joining together behind the anus. Mouth terminal, of medium diameter; buccal cavity broadly cylindrical; pharyngeal pores basal; intestine broadest at the rear mid-body and narrowing behind; anus ventral at U92. Hermaphroditic; possibly protandric, testes begin at the PhJIn, vasa deferentia run rearward, but termini not seen; female system not seen, nor were frontal or caudal organs.

##### Description:

**Figure 3.**
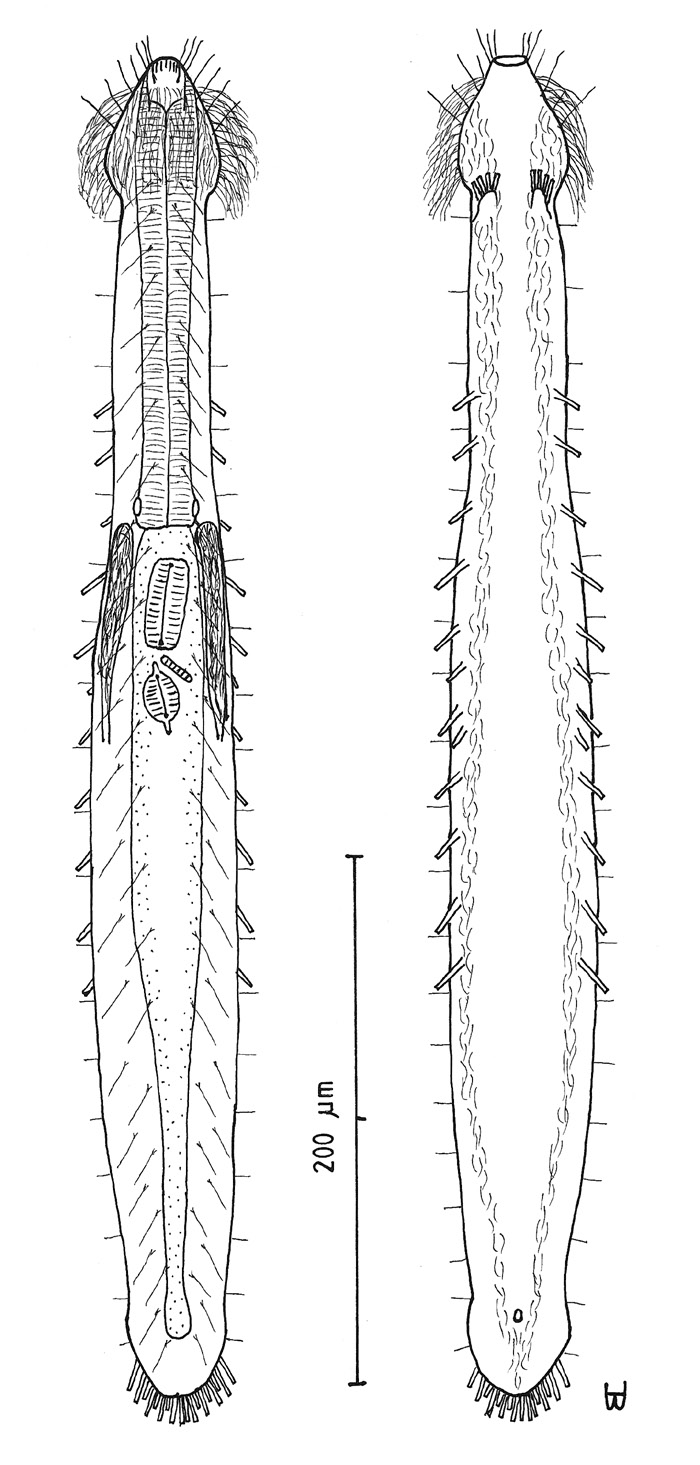
*Cephalodasys saegailus* sp. n. dorsal and ventral views of a mature adult (Lt=517, LPh=196 µm) from Sidi Abd al-Rahman, Egypt; dorsal with dorsal and lateral body cilia, digestive and reproductive tracts included, ventral with adhesive tubes and locomotor ciliary bands.

 Adult Lt 492–517 µm; LPh 191–196 µm to PhJIn at U39–U38 (Fig. 3). Body transparent, strap-shaped, dorsoventrally flattened, but vaulted dorsally; head pyriform, truncated apically, with a broad circumcephalic band of cilia at U02–U07, separated from the rest of the body by a long gradual neck constriction; trunk of medium width, sides of body broadest at the mid-body, and then narrowing behind to a rounded, slightly flaired caudum. Widths of head /neck /rear pharynx /midtrunk /caudal base /caudal flair, and locations along the length of the body are as follows: 43 /31 /36 /55 /36 /38 µm at U08 /U10 /U31 /U63 /U89 /U92, respectively. Cuticle is flexible; epidermis is finely granular; glands are lacking.

*Adhesive tubes:*TbA 4 per side (L 6–8 µm), thick, borne on broad fleshy asymmetrical hands that insert at U11–U12; TbVL 10 per side (L 10–16 µm), insert regularly from U24 to U66, three along the rear pharynx, the others along the fore- and midgut; TbV 2 per side (L 8–9 µm) occur amidst the TbVL series at U44 and U49; TbL/TbD absent; TbP 18 of varying lengths insert in more than one row around the rear of the rounded caudum, mostly projecting rearward (longer ones, L 8–10 µm, as # 1–2, 4, 6, 8–9 from either side, and shorter ones, L 5–7 µm, as # 3, 5, 7 from either side).

*Ciliation:* Three sensory hairs (L 7–15 µm) project forward on either side of the mouth and others (L 10–20 µm) project obliquely from the head; a broad band of cephalic cilia (L 15–20 µm) covers the head and joins the locomotor ciliature ventrally; 18–20 sensory hairs each occur regularly in lateral (L 4–6 µm), dorsolateral (L 10–14 µm) and dorsal (L 14–20 µm) columns along each side of the trunk. Ventral locomotor ciliature forms two longitudinal bands of short (L 4–6 µm), scattered cilia that follow the lateral body contours the entire length of the body, separate medially beneath the head and pharynx, but converging behind the anus and merging into a single band onto the caudum.

*Digestive tract:* Mouth terminal, but slightly inclined to the ventral surface, medium in width (10 µm diameter); buccal cavity semi-cylindrical, lightly cuticularized, having ca. 8 short longitudinal ridges set dorsally in the oral opening; pharynx of medium breadth, narrowest just before the well-developed, basal pharyngeal pores; intestine broad in front, narrowing behind; anus ventral at U92.

*Reproductive tract:* Hermaphroditic, probably protandrous; testes begin at the PhJIn (U34), with vasa deferentia continuing rearward past U50, but termini not seen; female system was not yet developed; frontal organ and caudal organ not seen.

*Ecology:* Sparse in frequency of occurrence (fewer than 10% of samples), scarce in abundance (3–5% of a sample); *sublittoral* in very fine to fine, well to medium-well sorted sand at 3 m water depth.

##### Geographical distribution:

 **MED:** *EGYPT* {Marsa Matruh [3-vid], ^Sidi Abd al-Rahman [vid]}.

##### Remarks:

 There are four video sequences of *Cephalodasys saegialus* sp. n., all from the eastern Mediterranean Sea in Egypt. These are available as MPEG 2 (and MPEG 1) from Hummon (2009): #755 a mature adult **Lectotype** of Lt=517 µm (LPh=196 µm), collected in June 1994 from Sidi Abd al-Rahman, Egypt; #754 a mature adult of Lt=492 µm (LPh=191 µm) from Marsa Matruh, Egypt; #753 a subadult of Lt=369 µm (LPh=158 µm) also from Marsa Matruh; and the other #752 a subadult of Lt=193 µm (LPh=95 µm) again from Marsa Matruh. *Cephalodasys saegialus* feeds on a diversity of diatoms. The largest specimen seen was a young adult and can be expected to reach a longer length, perhaps 600 µm.

##### Etymology:

 Saegailus (Greek: *sos* + *aigialos* = meaning ‘safe shore’) refers to its shallow sublittoral habitat along the southern Mediterranean being sheltered from the west wind (zephyr).

##### Taxonomic affinities:

*Cephalodasys saegialus* sp. n. is the only species in the genus that has the following combination of characters: a pyriform head, with a transverse band of cephalic cilia, and PhJIn at U39–U38, with TbA 4 per side; TbVL 10 per side at U24–U66, with three along the rear pharynx, the others along the fore- and midgut; TbV 2 per side, amidst the TbVL at U44 & U49; TbL *per se* /D absent; and TbP 18, of varying sizes, insert in more than one row around the rear of the caudum, with testes but neither ova nor accessory sex organs seen. *Cephalodasys saegialus* belongs in the group of species that have pyriform heads, but is unique in having an extended gap between TbL and TbP and in having TbV, compared with other species with which it might be compared, namely *Cephalodasys cambriensis* (Boaden, 1963); *Cephalodasys littoralis* Renaud-Debyser, 1964; *Cephalodasys pacificus* Schmidt, 1974; and *Cephalodasys turbanelloides* (Boaden, 1960).

### Family DACTYLOPODOLIDAE Remane, 1927 Genus Dactylopodola Strand, 1929

#### Dactylopodola agadasys

Hochberg, 2003

http://species-id.net/wiki/Dactylopodola_agadasys

[Dcp agds]

Figure 4

Dactylopodola agadasys  Hochberg, 2003: p. 41; Figs. 5–6. -- new species. Hummon et al. (2005: Tab. 1); Hummon (2009: N Am and E Med & Red Sea Databases); Hochberg (2008: p. 103; tab. 2; fig. 2A.)

##### Redescribed diagnosis:

 Adult Lt 390 µm; PhJIn at U32. Head bluntly rounded, but without ocelli, neck constriction extended but slight; trunk slender, with two broad caudal lobes that incise medially to U92, without a peduncle. Glands not seen; protonephridia 3 per side, at U32, U78 and U87; longitudinal muscles are striated. TbA 3 per side (L=6, 8, 11 µm) insert in parallel, protruding obliquely to the rear; TbVL 6 per side, arising in groups of 3/2/1 (L=14, 8, 6 / 17, 7 / 14 µm) at U36–U38 /U46–U49 /U57, all protruding obliquely to the rear, proceding rearward from longer to shorter in each group; TbP 6 per caudal lobe (L=8–10 µm), longest medially on each lobe. Mouth terminal, of medium breadth; buccal cavity goblet-shaped; pharynx width follows the head/neck contours, with inconspicuous basal pores that open well behind the neck constriction; intestine narrows fore to aft, anus ventral at U91. Ventral ciliation: a unified field beneath the head splits into a pair of longitudinal bands, each narrow in breadth, that continues rearward to the level of the anus, and a second pair of longitudinal bands that lie medially from U12 to U34, with a an isolated patch lying medially behind the anus. Probably parthenogenic; male system not seen; ovaries paired in hindgut region, with oocytes on both sides behind the predominant ovum that develops medially forward toward the midgut; caudal and frontal organs not seen.

##### Redescription:

**Figure 4.**
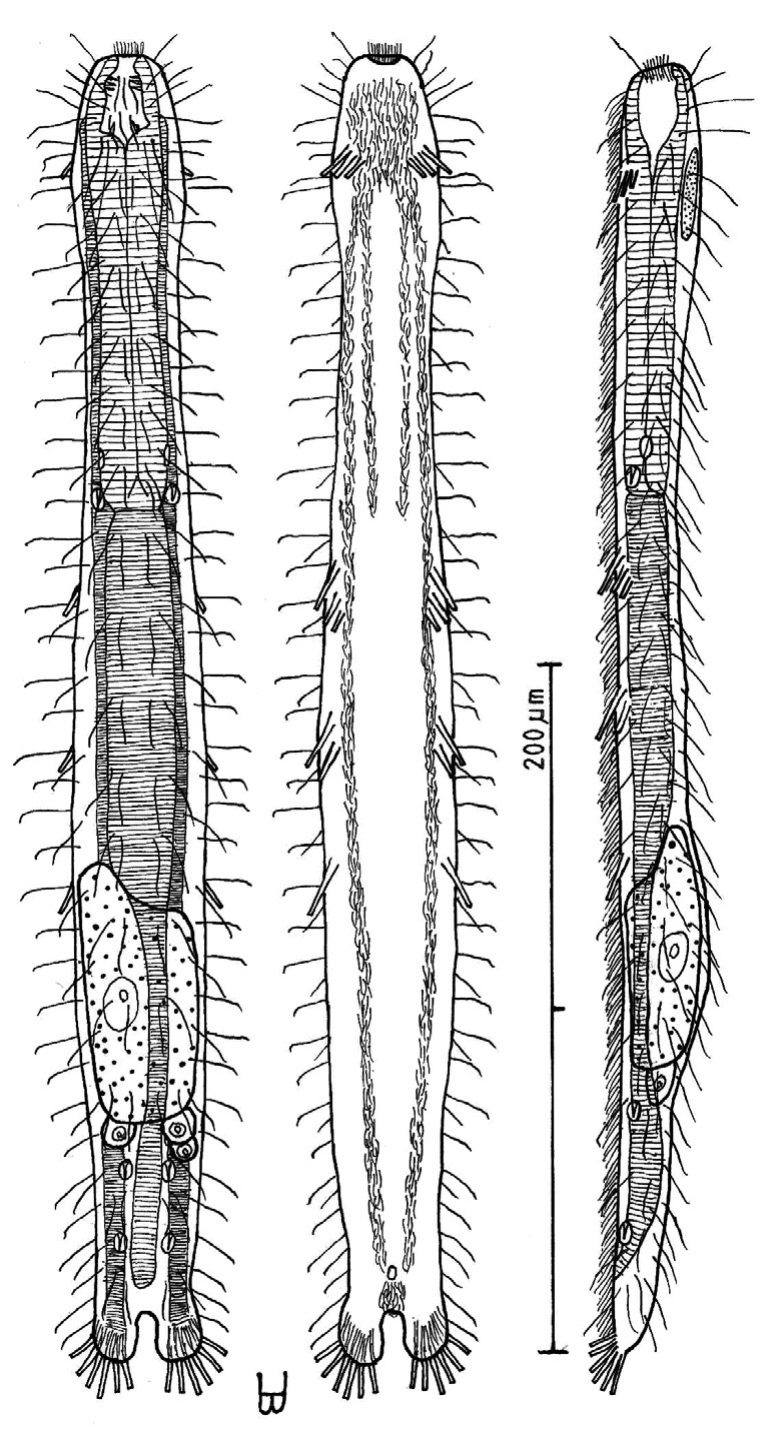
*Dactylopodola agadasys* Hochberg, 2003 dorsal, ventral and lateral views of a mature adult (Lt=390, LPh=126 µm) from Nabq, Egypt; dorsal with dorsal and lateral body cilia, digestive, reproductive and muscular tracts, and protonephridia; ventral with adhesive tubes and locomotor ciliary bands; lateral with digestive, reproductive and muscular tracts, adhesive tubes, and protonephridia.

 Adult Lt 322–390 µm; LPh 103–126 µm to PhJIn at U32–U33 (Fig. 4). Body flattened ventrally, vaulted dorsally, comprised of bluntly rounded head that lacks ocelli, neck constriction extended but slight; trunk slender, with two broad caudal lobes that incise medially to U92, without a peduncle. Widths of head /neck /trunk /caudal base are as follows: 30 /24 /36 /25 µm at U10 /U18 /U52 /U88, respectively. Glands absent. Protonephridia 3 per side, each with 2 flagellae, located just before the PhJIn at U32, and in the hindgut region at U78 and U87. Longitudinal muscles are striated, as is characteristic of members of this family.

*Adhesive tubes*: TbA 3 per side (L 6, 8, 11 µm), inserting in parallel at U05 and protruding obliquely to the rear, proceding rearward from longer to shorter; TbVL 6 per side, arising in groups of 3/2/1 (L 14, 8, 6 /17, 7 /14 µm) at U36–U38 /U46–U49 /U57, all protruding obliquely to the rear, proceding rearward from longer to shorter in each group; TbL *per se* /TbD /TbV are absent; TbP 6 per caudal lobe (L 8–10 µm), longest medially on each lobe.

*Ciliation*: Sensory hairs (L 9–18 µm) are abundant on the body, in 5 columns per side – ventrolateral, lateral, 2 dorsolateral and dorsal – of 18–20 per side each from U00 to U93, the tips of each being curled to the rear. Ventral ciliation: a unified field beneath the head splits into a pair of longitudinal bands, each narrow in breadth, that continues rearward to the level of the anus, and a second pair of longitudinal bands lie medially from U12 to U34, with a an isolated patch lying medially behind the anus.

*Digestive tract*: Mouth terminal, slightly inclined toward the ventral, of medium breadth (8 µm in diameter), goblet-shaped buccal cavity large, with 4 internal sensory hairs (L 2 µm) per side, 3 laterally and 1 posteriorly; pharynx broadest in the buccal region, with breadth following the body contours in the head and neck region, basal pharyngeal pores inconspicuous, opening well behind the level of the neck constriction (U27); foregut broad, midgut narrowing, hindgut broadening a bit before the anus, which occurs ventrally at U91; circular muscules sheath both the pharynx and the intestine, the former more heavily than the latter.

*Reproductive tract*: Probably parthenogenic; male system not seen; ovaries paired in hindgut region, with oocytes on both sides behind the predominant ovum (77 × 33 µm) which develops medially forward toward the midgut; caudal and frontal organs not seen.

*Ecology*: Occasional in frequency of occurrence (10–30% of samples), scarce to prevalent in abundance (3% to greater than 30% of a sample, sometimes a co-dominant **[cdom]**); *littoral* in fine-medium, medium-well sorted to very poorly sorted clean coralline sand at mean low water to low water spring, 0–15 cm sand depth.

##### Geographical distribution:

 **ANW:** *UNITED STATES:* *Florida* {Bahia Honda SW [video], Crandon Park Inside}. **RED:** *EGYPT:* {Sharm el-Arab Inside, Na’ama Bay N, Nabq S [4-videos], Sharm el-Naga **[cdom]** [video], Ras Nasrani [video]}. **CRB:** *PANAMA:* Bocas del Toro {Isla Colón Site 4} **IND:** *AUSTRALIA:* Queensland {^Macleay Island W (27°35'S, 153°21'E)}

##### Remarks

**:** There are six video records of *Dactylopodola agadasys*, five from three locations on the Red Sea coast of Egypt, and one from the Atlantic coast of Florida, US (see below). It was listed among the marine gastrotrichs for which videos were available by Hummon, Todaro & Evans 2005. Four of these are available as MPEG 2 (and MPEG 1) from Hummon (2009), all from Nabq S, near Ras Mohamed National Park, on the South Sinai, Egypt: #770 an adult of Lt=389 µm (LPh=121 µm), collected in July 1994, #769 an adult of Lt=369 µm (LPh=120 µm), #772 an adult of Lt=362 µm (LPh=120 µm), and the other #773 an adult of Lt=322 µm (LPh=103 µm).

I found this species in the Red Sea sites while on a Fulbright Senior Research Scholarship during 1994; thus my drawings and videos antedate by seven years its formal description by Hochberg, 2003. He had found it in eastern Australia during 2001, the species now having a much broader biogeographical range than just Austalia or the upper Red Sea. His recent report of the species from Panama (Hochberg 2008) not only further extends its range, but has implications for an original distribution that would date it back to a time prior to the raising of the Isthmus of Panama some 3 million years ago during the Pliocene. During this past summer (2010), while reviewing videos from Florida collections made during February 1991 by Hummon, Evans & Todaro, I discovered that a species, thought when collected in to be of unknown identity, was in fact *Dactylopodola agadasys*. It is shown in video #920 (though with unknown length).

**Figure 5.**
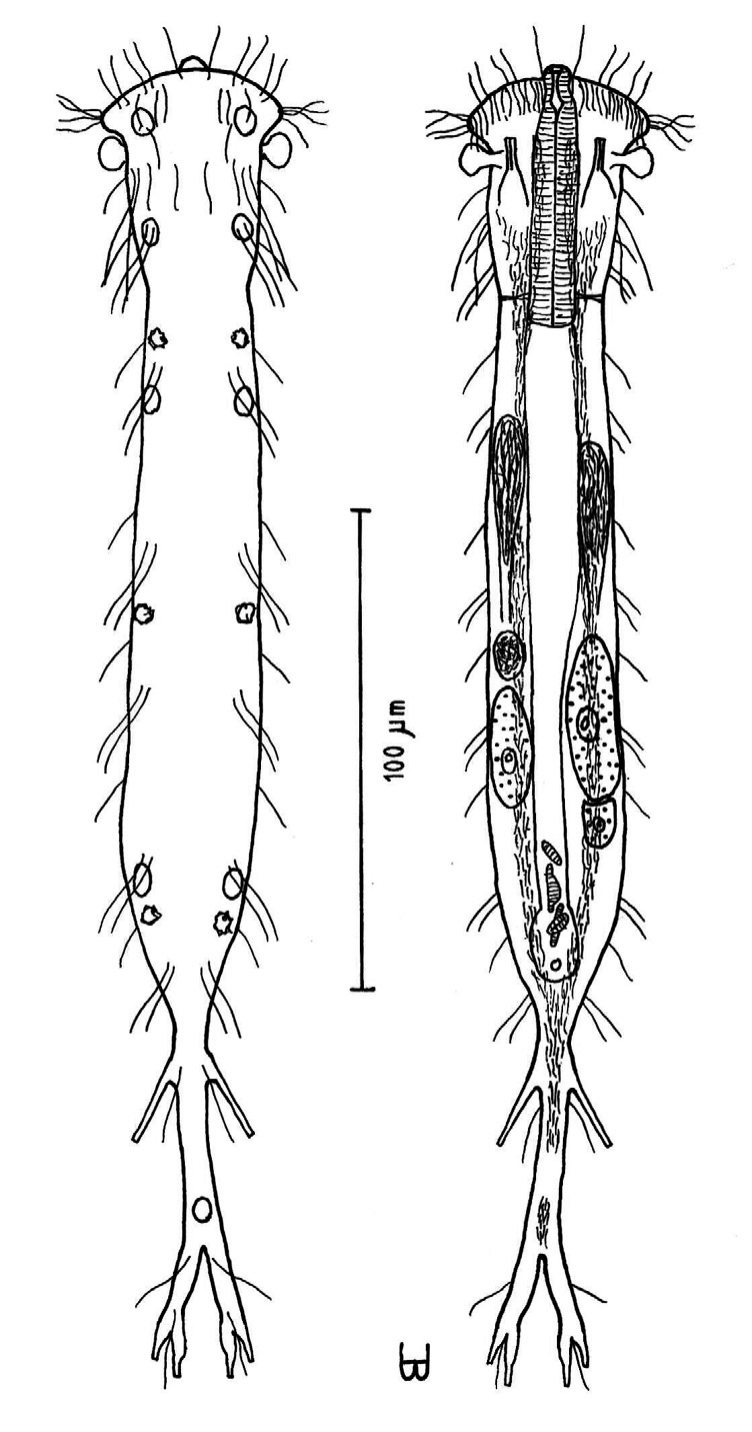
*Dendrodasys rubomarinus* sp. n. dorsal and ventral views of a mature adult (Lt=272, LPh=53 µm) from Giftun Island SE, near Hurghada, Egypt; dorsal with body conformation, dorsal and lateral body cilia and pattern of glands; ventral with digestive and reproductive tracts, pestle organs, adhesive tubes and locomotor ciliary bands.

**Figure 6.**
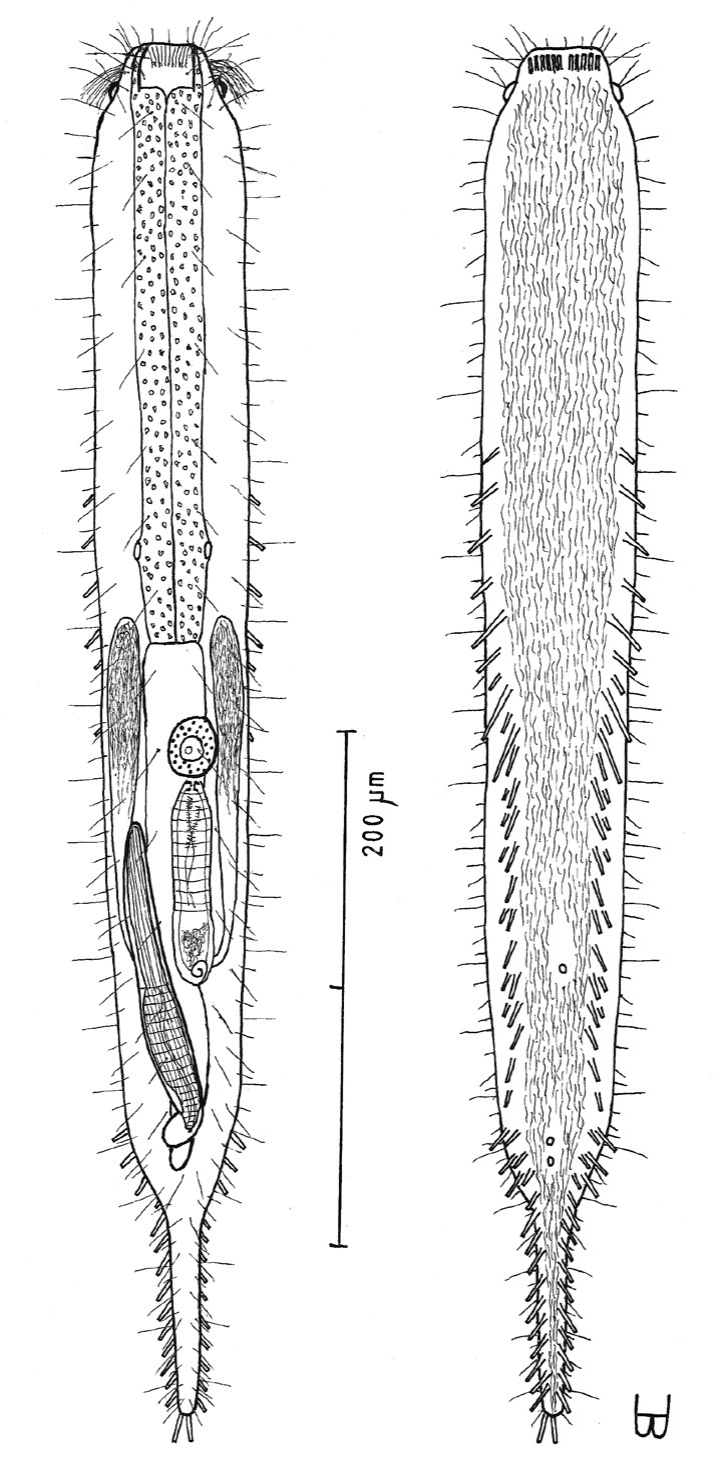
*Macrodasys imbricatus* sp. n. dorsal and ventral views of a mature adult (Lt=544, LPh=232 µm) from Main Beach, Ras Mohamed National Park, S. Sinai, Egypt; dorsal with dorsal and lateral body cilia, digestive and reproductive tracts; ventral with pestle organs, adhesive tubes and locomotor cilia.

The reason for redescribing this species is that Hochberg’s drawing (Hochberg 2003: Fig. 6) gives an incorrect impression of what the species looks like. By comparison with the photo presented in Hochberg Fig. 5, the drawing as presented in Fig. 6 is not sufficiently thin, and the TbP do not have attached cilia. Other difficulties with the drawing are that the TbA are of increasing length, interior to exterior, and not as shown; moreover the TbVL are grouped in a 3/2/1 sequence, all projecting obliquely to the rear, proceding rearward from longer to shorter in each group, and not occurring as a column of tubes that are equally spaced, though decreasing in size, as shown in Hochberg Fig. 6 (as is confirmed in one of several additional photographs given to me by Hochberg, see under this species in Gastrotrich Figures in Hummon 2009). I cannot speak to the ventral ciliation, as seen by Hochberg, but that which I have drawn in the redescription can be verified in the videos. I also can not speak to the identity of the specimen from Panama, the photo not giving sufficient detail for such an identification, but I give the author of the species the benefit of the doubt in his identification; I have myself seen the species from elsewhere in the Caribbean and made the same identification (as redescribed).

##### Etymology:

 Agadasys (Greek: *aga* + *dasys* = meaning ‘very hairy’) was named by Hochberg in reference to the numerous tactile cilia that cover the body.

##### Taxonomic affinities:

 *Dactylopodola agadasys* is presently the thinnest member of the genus, and the only species in the genus that has the following combination of characters: a bluntly rounded head and PhJIn at U33–U32, which also has TbA 3 per side in a parallel series that increases in length medial to lateral; TbVL 6 per side (in clusters of 3, 2 and 1, also parallel and increasing in length medial to lateral); and TbP 6 per side, radiating from broadly rounded lobes, but without TbL *per se* /TbD /TbV.

#### Genus Dendrodasys Wilke, 1954

##### Dendrodasys rubomarinus sp. n.

urn:lsid:zoobank.org:act:72488F13-99B8-4EC7-8C02-D38F1A52753B

http://species-id.net/wiki/Dendrodasys_rubomarinus

[Ddd rbmr]

Figure 5

Dendrodasys  EgyA Hummon (2009) [E Med & Red Sea Database].

###### Diagnosis:

 Adult 272 µm; PhJIn at U20. Body slender; head has crescent-shaped anterior, with protruding mouth and laterally directed lobes that have rounded tips, with a knob-shaped pestle organ on each side lying largely exposed beneath the rear of the head lobes; neck constriction slight, marking the pharyngeal pore openings; trunk parallel-sided, narrowing gradually in the rear, ending in a long, narrow caudal peduncle, with a bifurcate apex that indents medially to U90; pharynx short, pharyngeal pores basal; intestine narrows fore to aft; anus ventral at U68. Glands 7 per side, with another medially in the caudal peduncle; longitudinal muscles are striated. TbA 1 per side, with a duo-gland tube extending forward from a tapering base; TbL absent; TbP 3 per side, the longer one, arising from the caudal base, and the shorter two, arising from the bifurcate tip of the caudum. Mouth diameter narrow, protruding forward from anterior head curvature; small goblet-shaped buccal cavity moderately cuticularized; pharyngial pores located at the level of the neck constriction, and only detected with maturity; intestine broader in front, narrower behind, its lumen fringed by actively-beating cilia; anus ventral at U68. Locomotor ciliation forms paired longitudinal bands from the TbA rearward, joining behind the anus and continuing as a unified band onto the caudal peduncle, with a patch lying just before the bifurca. Hermaphroditic; paired testes lie along the fore-gut and paired ovaries along the rear mid-gut, eggs maturing rear to front; an ovoid frontal organ bearing active sperm occurs opposite the largest ovum on the right side.

###### Description:

 Adult Lt 244–272 µm; LPh 48–53 µm to PhJIn at U20 (Fig. 5). Body flattened ventrally, vaulted dorsally, comprised of head that is crescent-shaped anteriorly, with laterally directed lobes that have rounded tips and a protruding mouth, with a knob-shaped cephalic pestle organ (L 7 µm) on each side that lies largely exposed beneath the rear of the head lobes; neck constriction slight, marking the pharyngeal pore openings; neck constriction slight; the trunk is parallel-sided trunk, narrowing gradually in the rear, ending in a long, narrow caudal peduncle, with a bifurcate apex that indents medially to U90. Width of head /lobes /neck /trunk /caudal base are as follows: 28 /38 /21 /26–28 /7 µm at U08 /U04 /U18 /U31–U55 /U73, respectively. Glands are of two types, one with 4 per side, oval in shape (diam 4–6 µm) at U05 /U13 /U26 /U62 and a solitary medial gland on the caudal peduncle at U87, the other with 3 per side, ragged in shape and more refringent (diam 3 µm) at U21 /U42 /U65.

*Adhesive tubes*: TbA 1 per side (L 18 µm), comprising a long (6 µm) duo-gland tube extending forward from a heavy tapering base that inserts directly on the body surface, tubes being highly mobile and able to project laterally; TbL are absent; TbP 3 per side: one longer (L 12 µm) arising proximally from the caudal peduncle, and two shorter (L 5 & 9 µm) arising from the bifurcate tips of the 80 µm long pedunculated caudum.

*Ventral ciliation*: Head lobes bear a transverse row of cilia ventrally (L 8µm); head bears numerous cilia (L 10–25 µm) frontally, laterally and dorsally; sensory hairs 7 each per side occur in lateral (L 8–10 µm) and dorsal (L the first 26–28, others 14–16 µm) columns, lateral cilia occurring singly, dorsal cilia occurring in pairs, all spaced more or less evenly from U08 to U70, behind which are single lateral hairs on either side at U76 and U90 on the caudal peduncle. Locomotor ciliation runs in paired longitudinal bands from the TbA rearward, joining behind the anus and continuing as a unified band onto the caudal peduncle, with a patch lying just before the bifurca.

*Digestive tract*: Mouth diameter narrow (2 µm), protruding forward from the anterior head curvature; a goblet-shaped buccal cavity is moderately cuticularized; pharynx narrow, its basal pores located at the level of the neck constriction, and only detected with maturity; intestine narrow, broader in front, narrowing markedly half way along its length, but with a bulge around the anus, its lumen being fringed by actively-beating cilia; anus ventral at U68.

*Reproductive tract*: Simultaneous hermaphrodites; testes paired, but uneven in origin, along the fore-gut, its vasa deferentia extending rearward, but their termini not seen; sperm (L 25 µm) show half a spirally thickened head and half a flagellum; ovaries, with several (3–7) immature ovules, are located along the rear mid-gut, with the most mature ova developing medially forward between ovary and testes; an ovoid frontal organ bearing active sperm occurs opposite the largest ovum on the right side.

###### Ecology:

 Occasional (10–30% of samples) in frequency of occurrence, scarce to prevalent (3% to more than 30% of a sample, the latter sometimes a co-dominant **[cdom]**) in abundance; *littoral* in clean fine to coarse, well- to poorly sorted coralline sands at low water neap to low water spring; *sublittoral* in medium-fine to medium, well to medium sorted, clean coralline sands, often mixed with shell and coral gravel, at 1–5 m water depth.

###### Geographical distribution:

 **RED SEA:** *EGYPT*: {Sharm el-Arab Inside, Marsa Bareika N, ^Giftun Island SE (27°10'N, 33°57'E) **[cdom]** [video], Main Beach Ras Mohamed NP [2-videos], Nabq [video]}.

###### Remarks:

There are four video sequences of *Dendrodasys rubomarinus* sp. n., all from the Red Sea in Egypt. All four are available as MPEG 2 (and MPEG 1) from Hummon (2009): #797 a mature **Lectotype** adult of Lt=272 µm (LPh=53 µm), collected in June 1994 from Giftun Island SE, near Hurghada, Egypt; #800 a mature adult of Lt=258 µm (LPh=51 µm) from Main Beach Ras Mohamed National Park, S. Sinai, Egypt; #799 a mature adult of Lt=244 µm (LPh=48 µm) also from Main Beach; and the other #798 a subadult of Lt=152 µm (LPh=35 µm) from Nabq, Egypt, also on the S. Sinai. The hind-gut is often found bearing diatom frustules.

###### Etymology:

 Rubomarinus (Latin: *ruber* + *marinus* = meaning ‘red sea’) refers to the body of water in which it was found, the Red Sea.

###### Taxonomic affinities:

 *Dendrodasys rubomarinus* sp. n. is the only member of the genus with rounded head lobes and pestle organs that are knob-like, that also has the neck constriction occurring at the pharyngeal pores, rather than behind the pharyngeal pores, and has bi-lateral testes. Two of the other four species closely resemble *Dendrodasys rubomarinus*, but differ in detail: *Dendrodasys gracilis* Wilke, 1954 has rounded head lobes, pyriform pestle organs and bi-lateral testes, while *Dendrodasys affinis* Wilke, 1954 [see also Hummon et al. (1998)] has rounded head lobes, lobiform pestle organs, and uni-lateral testes.

### Family MACRODASYIDAE Remane, 1927 Genus Macrodasys Remane, 1924

#### Macrodasys imbricatus sp. n.

urn:lsid:zoobank.org:act:A51FDF9E-8A42-406D-B38A-BE6F770F5D47

http://species-id.net/wiki/Macrodasys_imbricatus

[Mcd imbr]

Figure 6

Macrodasys  EgyI Hummon (2009) [E Med & Red Seas Database].

##### Diagnosis:

Adult being described Lt 544 µm; PhJIn at U43. Head stepped, narrowing toward the mouth, pestle organs in the step at U03; trunk broadest in the pharyngeal region, narrowing gradually to the long caudum. Glands inconspicuous. TbA 5–6 per side, in transverse rows that insert directly on the body; TbL/TbV 35 per side, the series beginning as TbL, then rotating to TbV and finally along the caudal base the two series duplicating one another; TbL 7 per side, with 0 along the fore and and 4 along the rear pharynx, 2 along the rear intestine, and 1 behind the anus; TbV 28 per side, uneven in size and location, but symmetrical in placement, with 1 along the rear pharynx, and the remainder in the intestinal region, the final tubes duplicating those of the rear of the TbL series; TbP 11 per side, surrounding the elongate caudum. Locomotor ciliature: a single field runs from the oral opening to the tip of the caudum, unciliated in spots surrounding ventral openings. Mouth terminal, of medium-broad width; buccal cavity lightly cuticularized, expanding with depth; pharyngeal pores para-basal; intestine narrows gradually to the rear; anus ventral at U80. Hermaphroditic; testes begin just before the PhJIn, vasa differentia join beneath the frontal organ; ova probably develop rear to front; frontal organ tubular, with cuticular nozzle, lies behind the foremost ovum; long caudal organ with muscular/glandular construction in a half to half ratio, extensively overlapping with the frontal organ.

##### Description:

 Adult being described Lt 544 µm (others Lt 541–625); LPh 234 µm (others LPh 200–205) to PhJIn at U43 (others PhJIn at U32–U38) (Fig. 6). Body medium in length as an adult, ventrally flattened, dorsally vaulted; head stepped, narrowing toward the mouth, bearing pestle organs in the step at U03; trunk broadest in the pharyngeal region, narrowing gradually to the long caudum. Widths at pestle organs /pharynx /PhJIn /anus /caudum, and locations along the length of the body are as follows: 40 /60 /56 /31 /22–6 µm at U03 /U26 /U43 /U80 /U84–U98, respectively. Glands not conspicuous.

*Adhesive tubes*: TbA 5–6 per side (L 7 µm), in transverse rows, which insert directly on the postoral body surface at U02 and project forward; TbL/TbV are unusual and complex, with 35 per side, from U28 to U95, with the first 4 being lateral and the others rotating ventrally, while the last TbL 3 along the caudal base are duplicated by 3 of the last 4 TbV; TbL 7, symmetrically and evenly placed and of similar size, with 2 along the fore and 2 along the rear pharynx, 2 along the rear intestine, and 1 more behind the anal aperture; TbV 28 per side, symmetrically placed but uneven in spacing and size (L 6–18 µm), with 1 along the rear pharynx, and the remainder in the intestinal region, 2 of which occur behind the anus, with numerous cases of side-by-side tubes; TbP 11 per side (L 10–12 µm) symmetrically surround the elongate caudum.

*Ciliation*: Sensory cilia (L 5–12 µm) occur around and on either side of the mouth; head lacks a ciliary corona, other sensory cilia arise in four columns on either side of the body: lower lateral (L 12–15 µm), upper lateral (L 7–9 µm), dorsolateral (L 15–26 µm) and dorsal (L 15–26 µm), with about 19, 34, 27 and 16 per column. Ventral locomotor cilia form a single field that lies between the TbL/V series back to the tip of the tail, with bare spots surrounding the ventral reproductive and anal openings.

*Digestive tract*: Mouth terminal, of medium-broad width (16 µm diameter); inner mouth rim bears a series of longitudinal ridges, extending half the depth of the buccal cavity; buccal cavity is mug-shaped, expanding slightly from oral opening to base and, is lightly cuticularized; pharyngeal musculature can be seen, with para-basal pharyngeal pores at U36; intestine is broadest in front, narrowing slowly to the rear; anus is ventral at U80.

*Reproductive tract*: Hermaphroditic; testes begin just before the PhJIn and vasa deferentia extend back to the rear of the frontal organ, though their termini were not seen; ova probably develop from rear to front, but only one small ovum was seen (22 × 18 µm in size); a tubular frontal organ occurs behind the ovum, having a refractile cuticular nozzle and circular muscles in the front three-fifths, as well as containing motile sperm, and cellular material in the accessory chamber, covering the rear two-fifths; long caudal organ appears to have circular muscles over the rear half that lie atop longitudinal muscles, which occur over the entire length of the organ, except for its rearmost glandular sac that lies at an obtuse angle to the rest.

##### Ecology:

 Occasional in frequency of occurrence (10–30% of samples), scarce to prevalent in abundance (3 to greater than 30% of a sample; occasionally a co- **[cdom]** or dominant **[dom]**); *littoral* in fine to very coarse, well to very poorly sorted, silicious or coralaginous sand with small amounts of detritus, at mean low water to extreme low water, 0–15 cm depth, sometimes on a tombolo; *sublittoral* in fine to coarse, medium well to very poorly sorted sand or coral debris at 1–7 m water depth, sometimes in sand occupying a ‘blowout’ depression on a coral platform.

##### Geographical distribution:

 **RED SEA:** *EGYPT:*{16 km S of Ein Sukhna [video], Marsa Bareika W, Sharm el-Arab Inside [video], Hammam Pharoan **[dom]** [videos], ^Main Beach Ras Mohamed NP (27°44'N, 34°12'E) [video], Ras Nasrani, Tareef el-Reeh [video], Tip Ras Mohamed NP **[cdom]}**; *ISRAEL:* {Coral Beach M2 [video], M3 [3-videos], M4 [video], Coral Beach N)}

##### Remarks:

 There are ten video sequences of *Macrodasys imbricatus* sp. n., all from the upper Red Sea in Egypt and Israel. Four of these are available as MPEG 2 (and MPEG 1) in Hummon (2009): #1365 a mature adult of Lt=625 µm (LPh=200 µm) from Hamman Pharoan, Egypt; #1347 a mature **Lectotype** adult of Lt=544 µm (LPh=232 µm), collected in July 1994 from Main Beach Ras Mohamed National Park, S. Sinai, Egypt; #1364 a mature adult of Lt=541 µm (LPh=205 µm) also from Hamman Pharoan; and #1929 a subadult of Lt=242 µm (LPh=97 µm) from the Coral Reserve, Eilat, Israel.

##### Etymology:

 Imbricatus (Latin: *imbricatus* = meaning ‘overlapping’) refers to the extensive overlap between caudal and frontal organs.

##### Taxonomic affinities:

 *Macrodasys imbricatus* sp. n. is the only species in the genus with an stepped anterior, with pestle organs in the step, a long tail, and a PhJIn of U32–U43, which also has TbA 5–6 per side in transverse rows; a TbL formula of 7=0,4/2,1 (0 along the fore and 4 along the rear pharynx/2 along the rear intestine and 1 behind the anus); a TbV formula of 27=0,1/25,1; and TbP 11 per side; but without TbD. Two characters of *Macrodasys imbricatus* sp. n. are unique thus far in the genus: one is the rotation of the TbL series to a TbV position and the rearward duplication of the two; the other is the remarkable overlap between caudal and frontal organs. To be compared with *Macrodasys imbricatus* sp. n., a species would have to have a tubular frontal organ and an elongate caudal organ that overlaps it by more than half. While there are several with tubular frontal organs, few have overlapping caudal with frontal organs. Only two species may meet these criteria: *Macrodasys gylius* Hummon, 2010 and *Macrodasys neapolitanus* Papi, 1957. The former has many fewer TbL/TbV than in *Macrodasys imbricatus* sp. n. and those that are present do not rotate from TbL to TbV and back, while the latter has few TbL occurring in front of the caudum and no TbV.

#### Macrodasys macrurus sp. n.

urn:lsid:zoobank.org:act:E10547B7-8A68-4A19-9DDE-B4A53379831B

http://species-id.net/wiki/Macrodasys_macrurus

[Mcd mcur]

Figure 7

Macrodasys  EgyE Hummon (2009) [E Med & Red Seas Database].

##### Diagnosis:

 Adult Lt being described 590 µm; PhJIn at U36. Head ovoid, with a narrow band of circumcephalic cilia at U02 and pestle organs at U03; trunk broad throughout, narrowing gradually along the rear intestinal region to the long caudum. Glands inconspicuous. TbA 11 per side, in arcs that insert directly on the body; TbL 4–5 per side, with 1–2 along the fore and 0 along the rear pharynx, 3 in the rear intestinal region, and 0 behind the anus; TbD 4 per side, of similar size and spacing, in the rear half of the body from U53 to U75; TbV 17 per side, nearly even in size and spacing, with 3 along the rear pharynx, the remainder in the intestinal region, and 0 behind the anus; TbP 7 per side symmetrically along the long caudum. Locomotor ciliature: a single field from the TbA to the tip of the caudum, unciliated in spots surrounding ventral openings. Mouth terminal, of narrow width; shallow buccal cavity is lightly cuticularized, expanding with depth; pharyngeal pores sub-basal; intestine broadest in front, narrowing quickly along the mid- to hind-gut, then bending around the caudal organ; anus ventral at U83. Hermaphroditic; testes begin just before the PhJIn, vasa differentia join beneath the frontal organ; ova develop rear to front; frontal organ elongate ovate, its nozzle in close contact with the ovum, with a beak-like cavity lying on the left that has a ventral pore and an accessory chamber behind; short, thick caudal organ has spiral muscles throughout, except for the rearmost glandular sac, the fore half appearing glandular, the rear half bearing an internal canal; caudal organ barely overlapping the accessory cell of the frontal organ.

##### Description:

**Figure 7.**
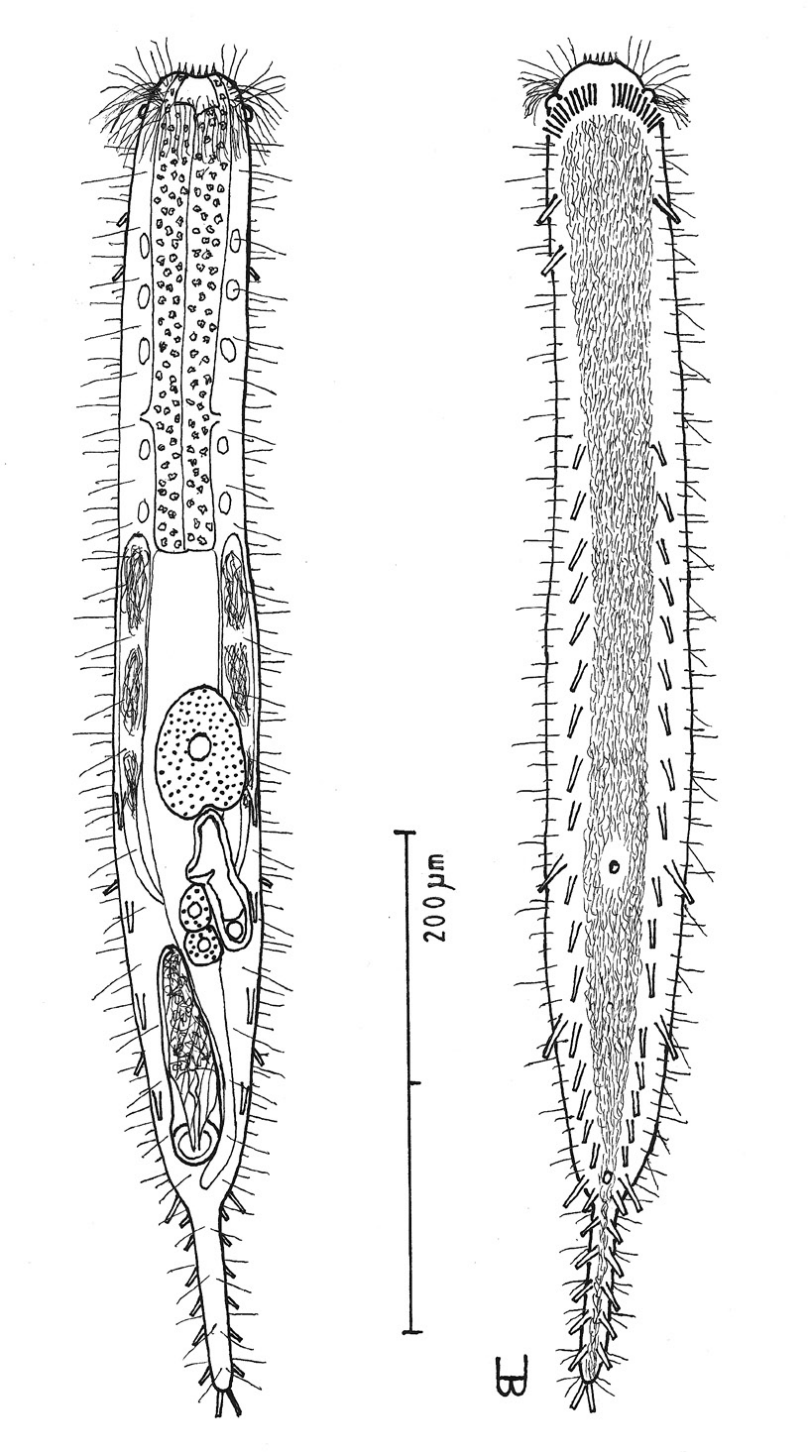
*Macrodasys macrurus* sp. n. dorsal and ventral views of a mature adult (Lt=590, LPh=192 µm) from the Giftun Village Spit Outside, near Hurghada, Egypt; dorsal with pestle organs, dorsal and lateral body cilia, digestive and reproductive tracts; ventral with adhesive tubes and locomotor cilia.

 Adult being described Lt 590 µm (others Lt 477–620); LPh 192 µm (others LPh 195–203) to PhJIn at U36 (others PhJIn at U33–U41) (Fig. 7). Body medium in length as an adult, ventrally flattened, dorsally vaulted; head ovoid, bearing a narrow band of circumcephalic cilia at U02 and pestle organs at U03; trunk broad throughout, narrowing gradually along the rear intestinal region to the long caudum. Widths at pestle organs /PhJIn /trunk /anus /caudum, and locations along the length of the body are as follows: 45 /51 /58 /24 /11–7 µm at U03 /U36 /U47 /U83 /U85–U98, respectively. Glands not conspicuous.

*Adhesive tubes*: TbA 11 per side (L 7–9 µm), in arcs that insert directly on the postoral body surface at U02 and project from forward to obliquely outward; TbL 4–5, of similar size, with 1–2 along the fore and 0 along the rear pharynx, 3 in the rear intestinal region, and 0 behind the anal aperture; TbD 4 per side, of similar size and spacing, all in the rear half of the body from U53 to U75; TbV 17 per side, nearly even in size and spacing (L 8–12 µm), with 3 along the rear pharynx, the remainder in the intestinal region, and 0 behind the anus; TbP 7 per side (L 9–10 µm) symmetrically along the caudum.

*Ciliation*: Sensory hairs (L 16–20 µm) occur on either side of the mouth and on the head; a band of cilia surrounds the forehead (L 13–15 µm) at U02; other sensory hairs arise in four columns on either side of the body: lower ventrolateral (L 9–12 µm), upper ventrolateral (L 9–12 µm), lateral (L 12–14 µm) and dorsolateral (L 20–24 µm), with about 30–32, 30–32, 28 and 24 per column. Ventral locomotor ciliature forms a single field that lies from the TbA back between the TbL/V series back to the tip of the tail, with bare spots surrounding the ventral openings.

*Digestive tract*: Mouth terminal, narrow (9 µm diameter), surrounded by sharp tooth-like projections; shallow buccal cavity expands from oral opening to its base and is lightly cuticularized; pharynx has sub-basal pharyngeal pores at U26; intestine is broadest in front, narrowing quickly at the mid- to hind-gut and bending around the caudal organ; anus is ventral at U83.

*Reproductive tract*: Hermaphroditic; testes beginning just before the PhJIn, containing pulses of sperm, and extending as vasa deferentia back to the rear of the frontal organ, though their termini were not seen; ova develop from rear to front, the largest (52 × 36 µm in size) lying above the rear fore-gut; frontal organ elongate ovoid, its nozzle in close contact with the ovum, with a beak-like cavity lying on the left that has a ventral pore, and an accessory chamber to the rear; sperm not seen internally; short, thick caudal organ has spiral muscles throughout, except for the rearmost glandular sac, the fore half appearing glandular, the rear half bearing an internal canal; caudal organ barely overlapping the accessory chamber of the frontal organ.

##### Ecology:

 Occasional in frequency of occurrence (10–30% of samples), scarce to prevalent in abundance (3 to greater than 30% of a sample; often a sub- **[sdom]**, co- **[cdom]**, or dominant **[dom]**); *littoral* in fine, well sorted to coarse-very coarse, poorly to very poorly sorted, coralline sand with small amounts of detritus, at low water neap to extreme low water, 0–15 cm depth; *sublittoral* in fine to coarse, medium well to very poorly sorted coralline sand and coral debris at 1–14 m water depth, sometimes between coral platforms or between patches of fringing reef.

##### Geographical distribution:

 **RED SEA:** *EGYPT:*{Marsa Bareika N **[sdom]**, Daghashland [video], Far Garden **[dom]** [video], Giftun Island SE **[cdom]** [3-videos]/SS, ^Giftun Village Spit Outside (27°10'N, 33°49'E) [video], Middle Garden, Moon Beach, Sharm el-Naga **[cdom]**, Sharm el-Sheikh, Tip Ras Mohamed NP [video]}.

##### Remarks:

 There are seven video sequences of *Macrodasys macrurus* sp. n., all from the upper Red Sea in Egypt. Five of these are available as MPEG 2 (and MPEG 1) from Hummon (2009): #1172 a mature adult of Lt=620 µm (LPh=203 µm) from Far Garden, S. Sinai, Egypt; #1167 a mature **Lectotype** adult of Lt=590 µm (LPh=207 µm), collected in June 1994 from Giftun Village Spit O, Egypt; #1173 a mature adult of Lt=477 µm (LPh=195 µm) from Daghashland, Egypt; #1170 a subadult of Lt=276 µm (LPh=122 µm) from Giftun Island SE, Egypt; and #1168 a juvenile of Lt=140 µm (LPh=73 µm) also from Giftun Island SE, Egypt.

##### Etymology:

 Macrurus (Greek: *macros* + *oura* = meaning long tail) refers to the appearance of a distinctly long tail, enhanced by the relative few TbP that it bears.

##### Taxonomic affinities:

 *Macrodasys macrurus* sp. n. is the only species in the genus with an ovoid head, a long tail, PhJIn at U33–U41, which also has 11 TbA per side in arcs; a TbL formula of 5=2,0/2,1 (2 along the fore and 0 along the rear pharynx/2 along the rear intestine and 1 behind the anus); a TbD formula of 4=0,0/4,0; a TbV formula of 17=0,3/14,0; and TbP 7 per side. There is no other species in the genus has a beaked frontal organ and a non-overlapping caudal organ, combined with columns of TbV and TbD, and a paucity of TbL.

#### Macrodasys nigrocellus sp. n.

urn:lsid:zoobank.org:act:522FAC42-E955-4EF9-A716-CA446B00E1C4

http://species-id.net/wiki/Macrodasys_nigrocellus

[Mcd ngoc]

Figures 8 9

Macrodasys  EgyF Hummon (2009) [E Med & Red Seas Database].

##### Diagnosis:

 Adult being described Lt 524 µm; PhJIn at U31. Head stepped, narrowing toward the mouth, with a band of circumcephalic cilia at U01–U02, pestle organs in the step at U03 and black ocelli borne just behind pestle organs at U05; trunk broader in the pharyngeal than in the fore-gut region, broadest in the mid-body region, narrowing quickly in the hind-gut region to the long caudum. Glands 8 per side. TbA 7–8 per side, in arcs that insert directly on the postoral body surface at U02–U03 and project forward to obliquely outward; TbL 14, of similar size, with 3 at U55, U64 and U73, and 12 at U80 and behind (7 along the hind-gut, and 4 behind the anal aperture); TbV 14 per side, even in size, but uneven in spacing, with 1 along the rear pharynx, 1 just behind the PhJIn and the remainder in the intestinal region from U41 to U77, mostly concentrated in the rear, and 0 behind the anus; TbP 10–11 per side symmetrically along the caudum. Locomotor ciliature: a single field lies between the TbV series from the TbA back onto the caudal base, with small bare spots surrounding the ventral openings. Mouth terminal, narrow; buccal cavity shallow, lightly cuticularized, expanding with depth; pharyngeal pores sub-basal; intestine narrows gradually front to rear; anus ventral at U84. Hermaphroditic; testes begin just before the PhJIn, vasa differentia join beneath the frontal organ; ova develop rear to front, with two large developing ova and several smaller bilaterally to the rear; frontal organ Y-shaped, its nozzle in close contact with the most developed ova, with an elongate cavity having a ventral pore lying on the left; caudal organ of medium length, its fore half appearing glandular, its rear half having an internal canal and a spiral muscular covering, except for the rearmost glandular sac, the caudal organ does not overlap the frontal organ.

##### Description:

**Figure 8.**
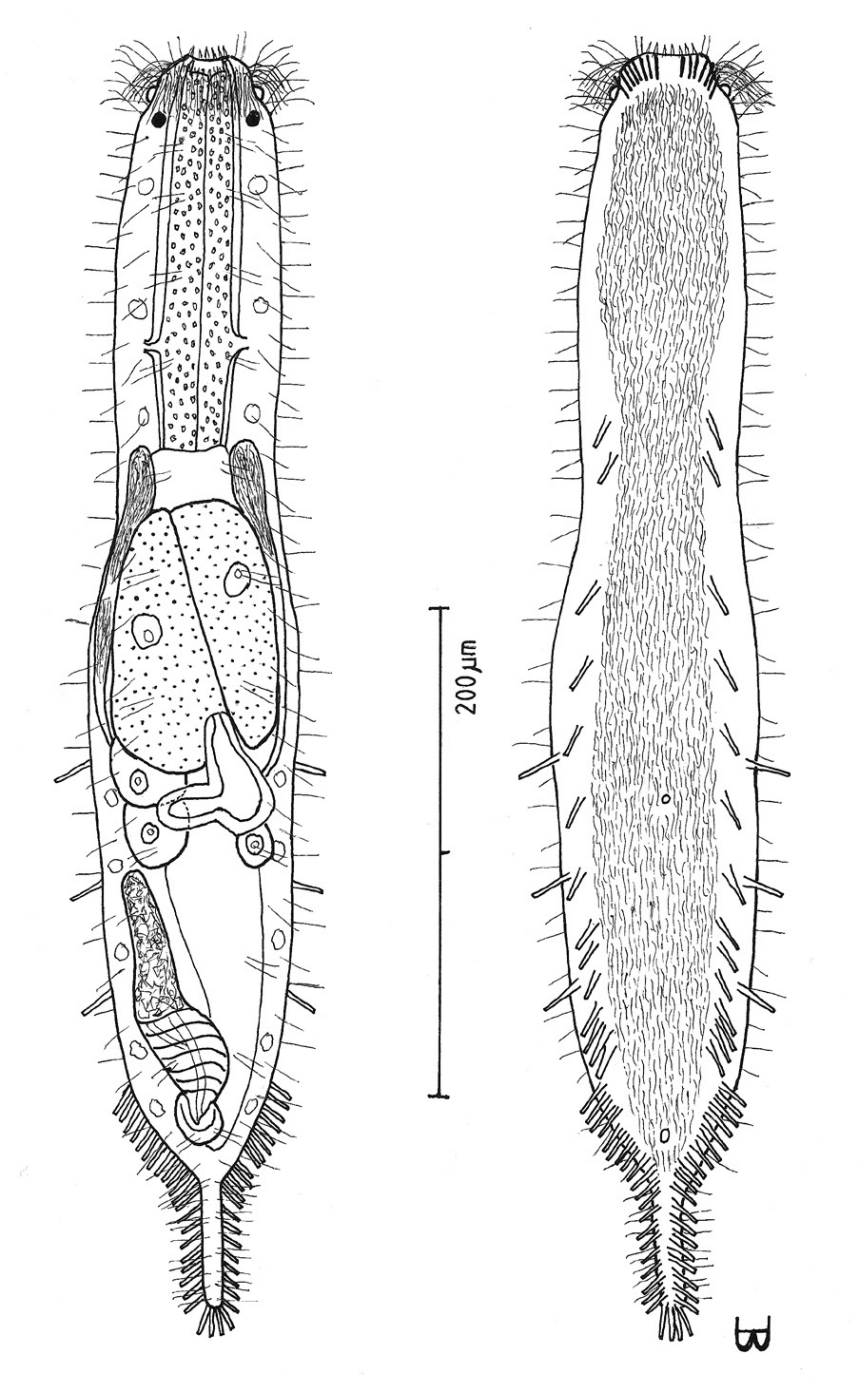
*Macrodasys nigrocellus* sp. n. dorsal and ventral views of a mature adult (Lt=300, LPh=161 µm) from Giftun Island SE, near Hurghada, Egypt; dorsal with pestle organs, dorsal and lateral body cilia, pattern of glands, and digestive and reproductive tracts; ventral with adhesive tubes and locomotor cilia.

 Adult being described Lt 524 µm (another Lt 558); LPh 161 µm (another LPh 230) to PhJIn at U31 (another to PhJIn at U41) (Fig. 8). Body medium in length as an adult, ventrally flattened, dorsally vaulted, robust; head stepped, narrowing toward the mouth, bearing a band of circumcephalic cilia at U01–U02, pestle organs in the step at U03 and black ocelli borne just behind pestle organs at U05 (black throughout its development from juvenile to adult); trunk broader in the pharyngeal than in the fore-gut region, broadest in the mid-body regions narrowing quickly in the hind-gut region to the long caudum. Widths at pestle organs /ocelli /PhJIn /trunk /anus /caudum, and locations along the length of the body are as follows: 41 /55 /64 /86 /34 /9–7 µm at U03 /U05 /U31 /U49 /U84 /U92–U97, respectively. Glands 8 per side (5 µm diameter), distributed 3 along the pharynx and 5 along the rear half of the intestine.

*Adhesive tubes*: TbA 7–8 per side (L 7–9 µm), in arcs that insert directly on the postoral body surface at U02–U03 and project from forward to obliquely outward; TbL 14, of similar size (L 12–18 µm), with 3 at U55, U64 and U73, and the other 11 at U80 and behind (7 along the hind-gut, and 4 behind the anal aperture); TbV 14 per side, even in size (L 12–15 µm) but uneven in spacing, with 1 along the rear pharynx, 1 just behind the PhJIn and the remainder in the intestinal region from U41 to U77, mostly concentrated in the rear, and 0 behind the anus; TbP 10–11 per side (L 12–15 µm), mostly symmetrical along the caudum, 2 at the terminus and 8–9 along the sides.

*Ciliation*: Numerous sensory hairs (L 10–16 µm) occur on either side of the head; a band of cilia surrounds the forehead (L 10–12 µm) at U01–U02; other sensory hairs (L 12–16 µm) arise in four columns on either side of the body: lateral, lower dorsolateral, upper dorsolateral, and dorsal (in pairs), with about 32, 24, 18 and 11 per column. Ventral locomotor ciliature forms a single field that lies between the TbV series from the TbA back onto the caudal base just behind the anus, but not beneath the caudum, with small bare spots surrounding the ventral openings.

*Digestive tract*: Mouth terminal, narrow in width (10 µm diameter), surrounded by sharp tooth-like projections; buccal cavity shallow, lightly cuticularized, expands from oral opening to its base; pharynx has sub-basal pharyngeal pores at U24; intestine is broadest in front, narrowing to the rear and bending around the base of the caudal organ; anus is ventral at U84.

*Reproductive tract*: Hermaphroditic; testes beginning just before the PhJIn, and extending as vasa deferentia back to the rear of the frontal organ where they join ventrally; ova develop from rear to front, with several large ova seen (two at 106 × 48 and 102 × 32 µm in size) and three smaller ovules bilaterally distributed to the rear; frontal organ Y-shaped, its nozzle in close contact with the most developed ovum, with an elongate cavern with its own ventral pore lying on the left, sperm not seen internally; caudal organ of medium length, its fore half appearing glandular, its rear half having an internal canal and a spiral muscular covering, except for the rearmost glandular sac, the caudal organ does not overlap the frontal organ.

**Figure 9.**
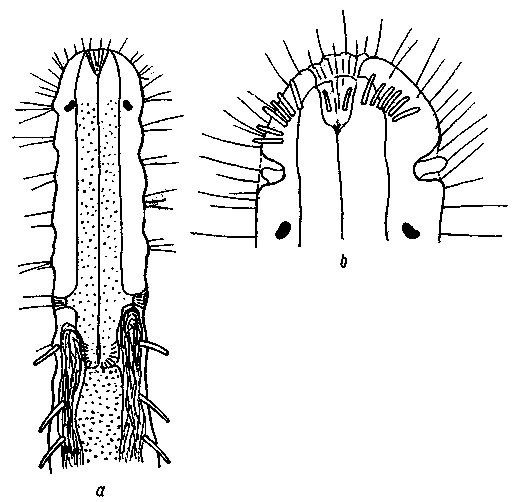
*Macrodasys* sp. Gerlach 1961 dorso-ventral and ventral views of the fore end of a specimen having black ocelli from Addu-Atol, the Maldive-Archipelago, Indian Ocean.

##### Ecology:

 Occasional in frequency of occurrence (10–30% of samples), rare to scarce in abundance (fewer than 1% to 5% of a sample); *littoral* in very fine, medium-well sorted coralline sand, at mean low water to extreme low water, 0–15 cm depth; *sublittoral* in very fine to very coarse, medium well to very poorly sorted coralline sand and coral debris at 1–4 m water depth, between patches of healthy or unhealthy fringing reef.

##### Geographical distribution:

 **RED SEA:** *EGYPT:*{Wadi ’Araba [video], ^Giftun Island SE (27°10'N, 33°57'E) [video], Moon Valley [3-videos], ’Uyun Musa [video]}; *ISRAEL:* {Snuba (Eilat) [video]} **INDIAN OCEAN:** *MALDIVES:* {Addu-Atoll}

##### Remarks:

 There are seven video sequences of *Macrodasys nigrocellus* sp. n., all from the upper Red Sea in Egypt and Israel. Four of these are available as MPEG 2 (and MPEG 1) from Hummon (2009): #1175 a mature adult of Lt=558 µm (LPh=230 µm) from Moon Valley, near Hurghada, Egypt; #1171 a mature **Lectotype** adult of Lt=524 µm (LPh=172 µm), collected in June 1994 from Giftun Island SE, also near Hurghada; #1178 a subadult of Lt=360 µm (LPh=154 µm) from the Wadi ’Arabah, Gulf of Suez, Egypt; and #1886 a juvenile of Lt=209 µm (LPh=104 µm) from the Snuba Dive Shop, Eilat, Israel.

##### Etymology:

Nigrocellus (Latin: *niger* + *ocellus* = meaning ‘black eye’) refers to the black ocelli that it bears.

##### Taxonomic affinities:

 *Macrodasys nigrocellus* sp. n. is the only species in the genus with a stepped anterior, with pestle organs in the step, black ocelli, a long tail, and a PhJIn of U31–U41, which also has TbA 7–8 per side in arcs; a TbL formula of 11=0,0/7,4 (0 along the fore and rear halves of the pharynx /7 along the rear intestine and 4 behind the anus), a TbV formula of 14=0,1/13,0 (mostly aggregated to the rear); and TbP 10 per side; but without TbD. There are three species that have mitten-shaped frontal organs: *Macrodasys caudatus* Remane, 1924 (though the specimen as originally described did not show a frontal organ), *Macrodasys pacificus* Schmidt, 1974 and *Macrodasys meristocytalis* Evans, 1994, none of which has black eye spots. No species yet described in this genus has black ocelli, but *Macrodasys* sp. Gerlach (1961: p.474, Fig. 3ab) from the Maldive Islands has such ocelli, and shows some characters that are similar (Fig. 9) to those of *Macrodasys nigrocellus* sp. n., namely the TbA, pestle organs and ocelli, though other characters such as the location of the testes and of the adhesive tubes make one wish that Gerlach had completed the entire drawing, rather than just the front half so that we might have seen the accessory reproductive organs.

#### Macrodasys scleracrus sp. n.

urn:lsid:zoobank.org:act:C0CDDFE1-9A05-4176-8A78-F62C37B17A88

http://species-id.net/wiki/Macrodasys_scleracrus

[Mcd scac]

Figures 10 11

Macrodasys  EgyG and ShmA Hummon (2009) [E Med & Red Seas, and Other Databases].

##### Diagnosis:

 Adult being described Lt 635 µm; PhJIn at U46. Head stepped, narrowing toward the mouth, with a narrow band of circumcephalic cilia at U02 and pestle organs in the step at U03; trunk of similar breadth throughout, narrowing quickly in the hind-gut region to the medium caudum. Glands inconspicuous. TbA 7–9 per side, in arcs that insert directly on the postoral body surface at U02–U04 and project forward to obliquely outward; TbL 11, of similar size, with 0 along the fore half and 2 along the rear half of the pharynx, 9 in the intestinal region, and 0 behind the anal aperture; TbV 14 per side, similar in size, but giving the impression of linear pairs, all in the intestinal region, with 0 behind the anus; TbP 11–12 per side symmetrically around the caudum. Locomotor ciliature: paired lateral bands lie between the TbV series back to the tip of the tail, with additional sparse cilia running medially to U73. Mouth terminal, of medium width; buccal cavity lightly cuticularized, expanding with depth; pharyngeal pores sub-basal; intestine narrows gradually front to rear; anus ventral at U91. Hermaphroditic; testes begin before the PhJIn, vasa differentia join beneath the frontal organ; ova develop rear to front, with a large ovum above the fore-gut and smaller ovules to the rear; frontal organ pyriform, its nozzle, having a thick refractive cuticular cap, and lying in close contact with the developing ova, bearing sperm internally; caudal organ of medium length, its fore half appearing glandular, its rear half having an internal canal and a spiral muscular covering, except for the rearmost sac; the caudal organ barely overlaps the rear of the frontal organ, and not at all in younger specimens.

##### Description:

**Figure 10.**
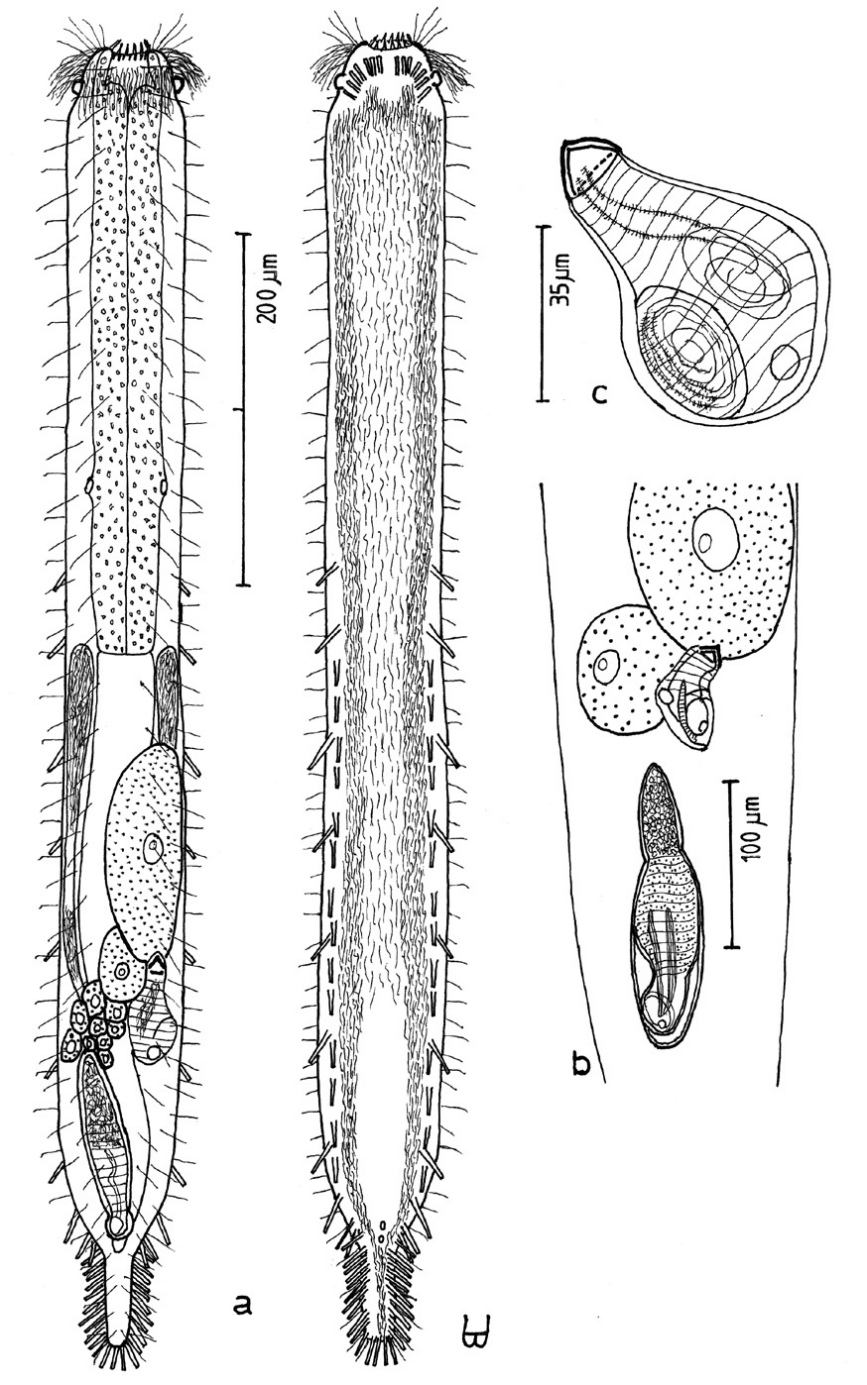
*Macrodasys scleracrus* sp. n. **A** dorsal and ventral views of a mature adult (Lt=635, LPh=290 µm) from Main Gate, Ras Mohamed National Park, S. Sinai, Egypt; dorsal with pestle organs, dorsal and lateral body cilia, digestive and reproductive tracts; ventral with adhesive tubes and locomotor ciliary bands **B** frontal organ with sperm from an animal of Lt=593 µm; C. reproductive organs from another animal of Lt=438 µm.

 Adult being described Lt 635 µm (others 394–800); LPh 290 µm (others 159–300) to PhJIn at U46 (others to PhJIn at U29–U47) (Fig. 10). Body medium in length as an adult, ventrally flattened, dorsally vaulted; head stepped, narrowing toward the mouth, with a narrow band of circumcephalic cilia at U02 and pestle organs in the step at U03; trunk of similar breadth throughout, narrowing quickly in the hind-gut region to the gradually delineated caudum of medium size. Widths at pestle organs /at U05 /PhJIn /anus /caudum, and locations along the length of the body are as follows: 45 /57 /54 /32 /13–10 µm at U03 /U05 /U46 /U91 /U92–U97, respectively. Glands inconspicuous.

*Adhesive tubes*: TbA 7–9 per side (L 7–9 µm), in arcs that insert directly on the postoral body surface at U02–U04 and project from forward to obliquely outward; TbL 11, of similar size (L 12–18 µm), with 0 along the fore half and 2 along the rear half of the pharynx, 9 in the intestinal region, and 0 behind the anal aperture; TbV 14 per side, similar in size (L 7–8 µm), but giving the impression of linear pairs, all in the intestinal region, with 0 behind the anus; TbP 11–12 per side (L 9–12 µm) symmetrically around the caudum.

*Ciliation*: Sensory hairs (L 10–20 µm) occur on either side of the mouth; a band of cilia surrounds the forehead (L 24–26 µm) at U02; other sensory hairs (L 8–16 µm) arise in three columns on either side of the body: lateral, dorsolateral, and dorsal, with about 30 per column. Ventral locomotor ciliature forms paired lateral bands that lie between the TbV series from the TbA back to the tip of the caudum, with additional cilia running in the medial space back to U73.

*Digestive tract*: Mouth terminal, of medium width (16 µm diameter), surrounded by sharp tooth-like projections; buccal cavity expands from oral opening to base and is lightly cuticularized; pharynx is covered by circular muscles (visible under DIC) and has sub-basal pores at U36; intestine is broadest in front, narrowing to the rear and bending around the base of the caudal organ; anus is ventral at U91.

*Reproductive tract*: Hermaphroditic; testes beginning just before the PhJIn, and extending as vasa deferentia back to the rear of the frontal organ, the termini not seen, though sperm can be seen in the vasa deferentia lateral to the frontal organ; ova develop from rear to front, with a large developing ovum (104 × 37 µm in size) above the fore-gut and ten smaller ovules to the rear; frontal organ asymmetrically pyriform (Fig. 10 a-c), its nozzle having a thick refractive cuticular cap, and lying in close contact with the largest developing ovum, and bearing sperm internally; caudal organ (Fig. 10 a) is of medium length, its fore half appearing glandular, and reducing in size proportionately with age (Fig. 10 c), its rear half having an internal canal and a spiral muscular covering, except for the rearmost sac; caudal organ barely overlaps the rear of the frontal organ, and not at all in older specimens.

*Ecology*: Common in frequency of occurrence (30–60% of samples), scarce to prevalent in abundance (3% to greater than 30% of a sample (often a sub- **[sdom]**, co- **[cdom]**, or dominant **[dom]**); *littoral* in fine, medium sorted to very fine-very coarse, poorly sorted silicious to coralline sand, with coral debris, at mean low water to extreme low water, 0–15 cm depth, occasionally occurring on a tombolo; *sublittoral* in fine, medium-well sorted to very fine-very coarse, very poorly sorted silicious to coralline sand, with coral debris at 1–15 m water depth, sometimes occurring in troughs and bars, between patches of healthy or unhealthy fringing reef, between coral platforms or in depressions in coral platforms.

**Figure 11.**
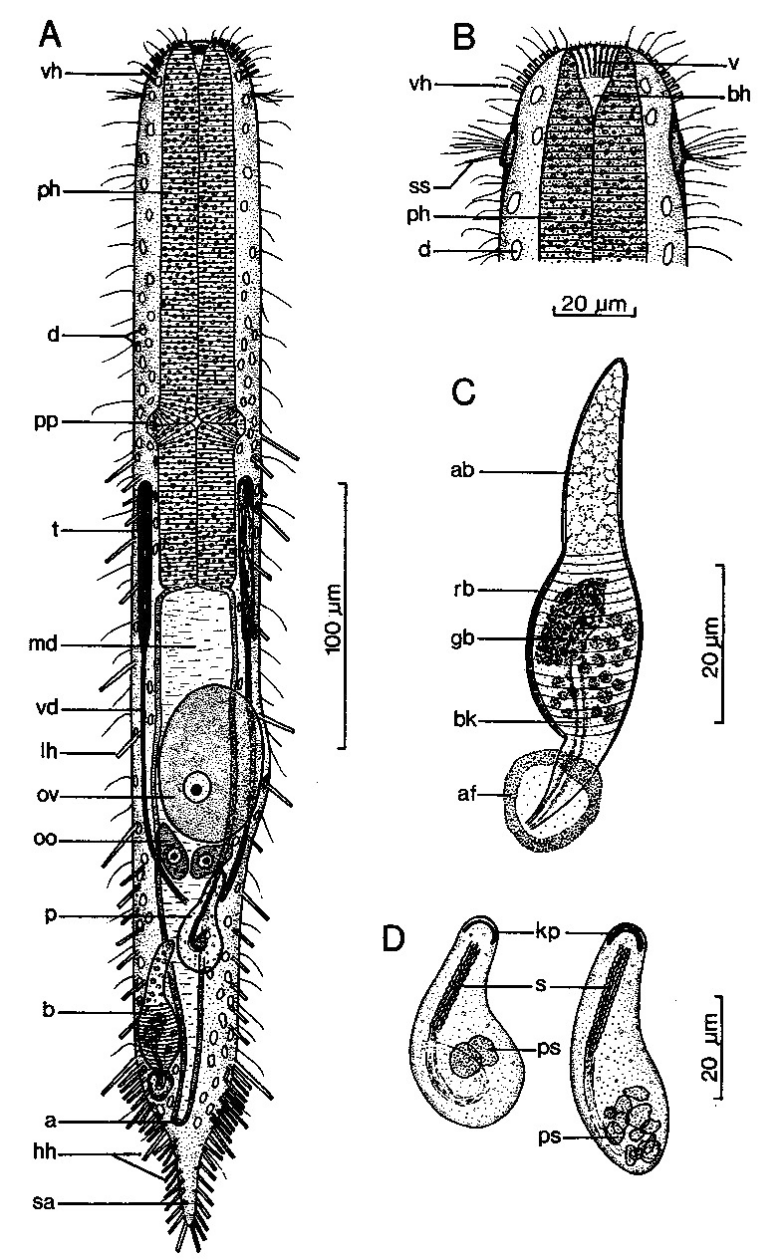
*Macrodasys* sp. A Schmidt, 1974 **A** habitus view of a mature adult (Lt=453, LPh=204 µm) from one of the three islands in the Galapagos Islands on which it was found, with pestle organs, body cilia, glands, digestive and reproductive tracts, and adhesive tubes **B** dorsal view of the fore end **C** caudal organ; and **D** two developmental stages of the frontal organ.

##### Geographical distribution:

 **MED SEA:** *EGYPT* {Betash ’Agami [video], Marsa Matruh, Sidi Abd al-Rahman [video]}. **RED SEA:** *EGYPT:*{Abu Ramada 1, Abu Ramada 2 [2-videos], Abu Ramada 3, Sharm el-Arab Outside, Wadi ’Araba, Marsa Bareika N, ^Marsa Bareika W **[dom]** [video], Daghashland, Ein Sukhna, Giftun Island SE [video], Giftun Island SS **[sdom]**, Main Beach Ras Mohamed NP [video], Mugawish **[dom]**, Na’ama Bay N **[dom]**, Na’ama Bay S [3-videos], Nabq **[dom]**, Nabq S **[sdom]** [3-videos], Sharm el-Naga **[cdom]**, Ras Nasrani, Nuweiba, Princess, Ras Qanti, Safaga, Ras Sudr [video], Tareef el-Reeh, ’Uyun Musa, West Gate Ras Mohamed NP **[dom]** [video]}; *ISRAEL:* {Coral Beach M3 [3-videos] & M4, Coral Beach S [video], North Beach [2-videos], Snuba DS} **PACIFIC NE:** *HAWAIIAN ISLANDS:* {Oahu: Hālona, Ka’a’awa [2-videos], Kaiona **[sdom]** [video], Penalu’u [video]}

##### Remarks:

 There are 25 video sequences of *Macrodasys scleracrus* sp. n. most from the upper Red Sea in Egypt and Israel, but some as well from the eastern Mediterranean Sea and from Oahu in the Hawaiian Islands. Ten of these are available as MPEG 2 (and MPEG 1) from Hummon (2009), seven from Egypt and three from Hawaii (collected by the author in 1994 and 2000/2003, respectively): from Egypt are #1320 a mature adult of Lt=800 µm (LPh=300 µm) from Abu Ramada, near Hurghada; #1315 a mature **Lectotype** adult of Lt=635 µm (LPh=207 µm), collected in July 1994 from Nabq S, S. Sinai; #1314 a mature adult of Lt=540 µm (LPh=159 µm) from West Gate, Ras Mohamed NP, S. Sinai; #1311 a mature adult of Lt=438 µm (showing reproductive organs) from Na’ama Bay, S. Sinai; #1316 a barely mature adult of Lt=394 µm (LPh=161µm) also from Nabq S; #1319 a subadult of Lt=215 µm (LPh=110µm) also from Abu Ramada, near Hurghada; and #1321 a juvenile of Lt=135 µm (LPh=70 µm) from Ras Sudr. From Hawaii are #2550 a mature adult of Lt=553 µm (LPh=262 µm) from Ka’a’awa Beach, Oahu; #2565 a subadult of Lt=407 µm (LPh=213 µm) from Penaluu Beach, Oahu; and #2551 a juvenile of Lt=135 µm (LPh=70 µm) from Ka’a’awa Beach, Oahu. This is the only new species of *Macrodasys* that occurs both on the Red Sea (IndoPacific Ocean) side and on the Mediterranean Sea side of the Suez Canal; it might be a Lessepsian migrant, since each of its Mediterranean occurrences occurs west of the openings of the Suez Canal, the direction of prevailing currents.

##### Etymology:

 Scleracrus (Greek: scleros + akros = meaning ‘hard, tough summit, peak’) refers to the refringent cuticular nozzle cap on the frontal organ.

##### Taxonomic affinities:

 *Macrodasys scleracrus* sp. n. is the only species in the genus with a stepped anterior, with pestle organs in the step, a medium tail, and a PhJIn of U29–U47, which also has TbA=7–8 per side in arcs; a TbL formula of 11=0,2/9,0 (0 along the fore half and 2 along the rear half of the pharynx/9 along the rear intestine and 0 behind the anus); a TbV formula of 14=0,0/14,0; and TbP=11–12 per side, with TbD absent.

Several species have frontal organs that are tipped by a small cuticular nozzle: *Macrodasys achradocytalis* Evans, 1994; *Macrodasys cunctatus* Wieser, 1957; *Macrodasys fornerisae* Todaro & Rocha, 2004; *Macrodasys gerlachi* Papi, 1957; *Macrodasys imbricatus* sp. n., *Macrodasys syringodes* Hummon, 2010; and *Macrodasys thuscus* Luporini, Magagnini & Tongiorgi, 1973, but only *Macrodasys* sp. A of Schmidt (1974: Figs. 5, 6) shows an animal that has a full cuticular cap (Schmidt: Fig. 6D, not Fig. 5D). Todaro (personal communication) has seen specimens from Santa Cruz, one of the Galapagos islands from which Schmidt reported *Macrodasys* sp. A., which he holds “can without a doubt be attributed to that species”, notwithstanding that Schmidt erred in his contention that testes originated well forward in the pharygeal region rather than at the pharynx-intenstinal junction where they usually occur. Todaro holds that *Macrodasys scleracrus* cannot be Schmidt’s *Macrodasys* sp. A because of differences in the shape of the caudal organ and the shape of the nozzle of the frontal organ, and differences between the two species with regard number and arrangment of the adhesive tubes of the TbL series. While I have not seen specimens from Schmidt’s collecting areas, I note that his TbL arrangement is confused by the composite habitus drawing of Schmidt that does not distinguish dorsal from ventral features, that the caudal organ in *Macrodasys scleracrus* sp. n. is somewhat variable, and that the new species was found several times on Oahu in the Hawaiian Islands. In my estimation, the reproductive similarities of the frontal organ outweighs other differences, and argues that *Macrodasys* sp. A may be a variant of *Macrodasys scleracrus* sp. n.

#### Genus Urodasys Remane, 1926

##### Urodasys toxostylis sp. n.

urn:lsid:zoobank.org:act:7F23E58F-935D-41F7-AD00-C3F84BDFF483

http://species-id.net/wiki/Urodasys_toxostylis

[Urd txst]

Figure 12

Urodasys  EgyA Hummon (2009) [E Med & Red Seas Database].

###### Diagnosis:

 Trunk of adult specimen being described Lt 440 µm, Lt tail 1100 µm; PhJIn at U40 of the trunk. Head bluntly ovate, with a narrow band of circumcephalic cilia and broad pestle organs at U05; neck constriction slight; trunk broadest along the mid-gut, narrowing gradually in the hind-gut region to the elongate tail. Glands 15–16 per side. TbA 7 per side, 3 in transverse rows at U07, which project obliquely forward, and 4 in longitudinal columns at U08–U17, which project obliquely outward, all inserting directly on the postoral body surface; TbL 4 per side, similar in size, all in the intestinal region; TbVL 4 per side, similar in size, 2 along the fore and 0 along the rear pharynx, and 2 in the rear intestinal region; TbD 5 per side, similar in size, 1 along the fore and 0 along the rear pharynx, and 4 in the intestinal region; TbV absent; TbP 10 or more per side, asymmetrical along the tail, depending on its length. Locomotor ciliature: paired lateral bands run from the TbA back to the rear of the caudal organ, with more sparse cilia medially from the TbA to U49. Mouth terminal, narrow; buccal cavity lightly cuticularized, expanding with depth; pharyngeal pores sub-basal; intestine narrowing gradually front to rear, lacking an anus. Probably hermaphroditic, though testis was not seen; ova develop from rear to front, with two bilateral ova in the mid-gut region of recently mature specimens, much larger in fully mature specimens (video #1904); frontal organ not seen; caudal organ, appearing enclosed in an oval capsule, has a hyaline bulblet in the rear, with an internal canal, that cycles around to include a darkish mass on the left and a stylet on the right, which is curved in the rear, widening and straightening toward the front, where it has an asymmetrical bulb that has a symmetrical opening at the end.

###### Description:

**Figure 12.**
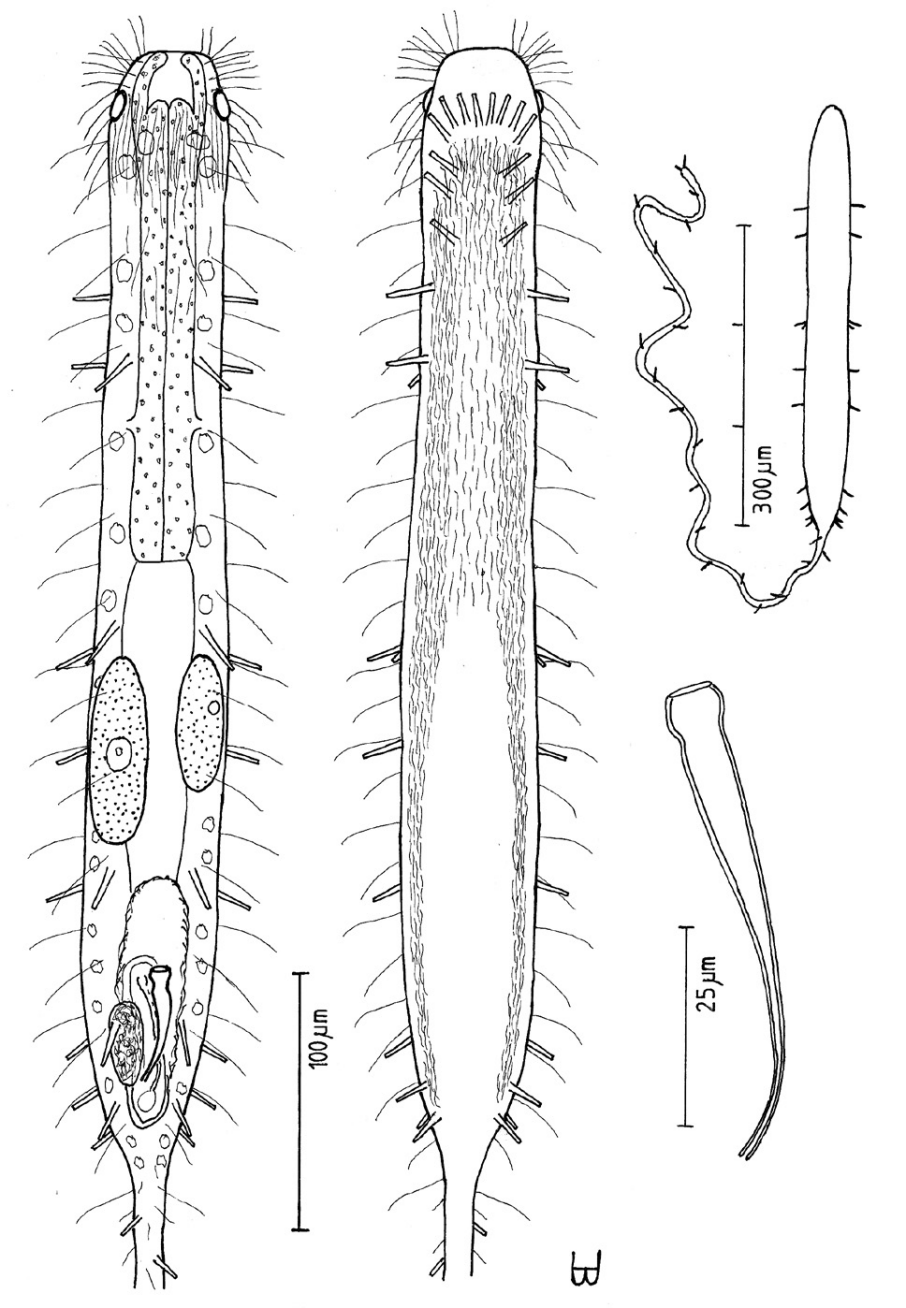
*Urodasys toxostylus* sp. n. **A** habitus of a mature adult (L trunk=440, LPh=174, L tail=1100 µm) from Giftun Island SS, near Hurghada, Egypt, showing relative sizes of trunk and tail **B** dorsal and ventral views of the same specimen; dorsal with pestle organs, dorsal and lateral body cilia, digestive and reproductive tracts, and adhesive tubes; ventral with adhesive tubes and locomotor ciliary bands **C** the stylet, magnified.

 Trunk of adult specimen being described Lt 440 µm (others Lt 413–480); Lt tail 1100 µm (others Lt 588–1620); LPh 174 µm (others LPh 161–173) to PhJIn at U40 (others PhJIn at U34–U40) (Fig. 12 A, B). Trunk medium in length as an adult, ventrally flattened, dorsally vaulted; head bluntly ovate, with a narrow band of circumcephalic cilia and broad pestle organs at U05; neck constriction slight; trunk broadest along the mid-gut, narrowing gradually in the hind-gut region to the elongate tail. Widths at pestle organs /pharyngeal pores /PhJIn /front, rear of caudal organ, and locations along the length of the body are as follows: 46 /48 /50 /54,30 µm at U03 /U33 /U46 /U72,97, respectively. Glands 15–16 per side (4–7 µm), larger in front than behind.

*Adhesive tubes*: TbA 7 per side (L 12 µm), 3 in transverse rows at U07, which project obliquely forward, curving laterally to form longitudinal columns of 4 at U08–U17, the latter projecting obliquely outward, all inserting directly on the postoral body surface; TbL 4 per side, similar in size (L 15 µm), all in the intestinal region at U54, U62, U75 and U88; TbVL 4 per side, similar in size (L 14–16 µm), 2 along the fore and 0 along the rear pharynx, and 2 in the rear intestinal region as the caudal base narrows; TbD 5 per side (also L 15–20 µm), 1 along the fore and 0 along the rear pharynx pores, and 4 in the intestinal region; TbV absent; TbP 10 or more per side (L 10–12 µm) asymmetrically about the tail, depending on its length.

*Ciliation*: Numerous sensory hairs (L 10–16 µm) occur around the head; a band of cilia surrounds the forehead (L 28–30 µm) at U05; other sensory hairs (L 25 µm) arise in two columns on either side of the body: lateral and dorsolateral, with about 30 per column. Ventral locomotor ciliature forms paired lateral bands that run from the TbA back to the rear of the caudal organ, with more sparsely placed cilia filling the space between from TbA to U49.

*Digestive tract*: Mouth terminal, narrow (6 µm diameter); buccal cavity expands from oral opening to base and is lightly cuticularized; pharynx has sub-basal pharyngeal pores at U33; intestine is broadest in front, narrowing to the rear, but lacks an anus.

*Reproductive tract*: Probably hermaphroditic, though testis was not seen; ova develop from rear to front, with two bilateral ova (74 × 24 and 55 × 19 µm in size) in the mid-gut region of recently mature specimens, and a much larger ovum (138 × 98 µm in size) appears in fully mature specimens (video #1904); frontal organ not seen; caudal organ, appearing enclosed as an oval capsule, has a hyaline bulblet in the rear, with an internal canal, that circles around to include a darkish mass on the left and a curved stylet on the right (Fig. 12 C), which is thin and curved in the rear, widening and straightening toward the front, where it has an asymmetrical bulb that has a symmetrical opening at the end.

###### Ecology:

 Occasional in frequency of occurrence (10–30% of samples), scarce to prevalent in abundance (3% to greater than 20% of a sample, sometimes a sub- dominant **[sdom]**); occasionally *littoral* in fine, medium-well sorted silicious sand, at mean low water to extreme low water, 0–15 cm depth; mostly *sublittoral* in fine, medium well sorted to very fine-very coarse, very poorly sorted silicious to corraline sand, with coral debris at 1–14 m water depth, sometimes in troughs, between patches of fringing reef, between coral platforms, in depressions in coral platforms or at the base of seagrass beds.

###### Geographical distribution:

 **RED SEA:** *EGYPT:*{Marsa Bareika N, Abu Ramada 1, Sharm el-Arab **[sdom]**, Daghashland, ^Giftun Island SS [video], Main Beach Ras Mohamed NP, Middle Garden [video], Mugawish, Na’ama Bay S [video], Nabq S, Sharm el-Sheikh, Ras Sudr, Tareeh el-Reeh S, ’Uyun Musa};*ISRAEL:* {Coral Beach M2, M3 [2-videos] & M5, Coral Beach N1 [2-videos], Princess Hotel [video]}.

###### Remarks:

 There are eight video sequences of *Urodasys toxostylis* sp. n., all from the upper Red Sea in Egypt and Israel. Six of these are available as MPEG 2 (and MPEG 1) from Hummon (2009): #1904 a mature adult of L trunk=480 µm (LPh=164, L tail=1200 µm) from the Coral Reserve (Eilat), Israel; #931 a mature adult of L trunk=438 µm (LPh=174, L tail=972 µm) from Na’ama Bay, S. Sinai, Egypt; #932 a mature **Lectotype** adult of L trunk=430 µm (LPh=173, L tail=1100 µm), collected in June 1994 from Giftun Island SE, near Hurghada, Egypt; #930 a mature adult of L trunk=413 µm (LPh=161, L tail=590 µm) from Middle Garden, S. Sinai, Egypt; #1909 a subadult of L trunk=387 µm (LPh=152, L tail=1620 µm) also from the Coral Reserve (Eilat); and #1923 a juvenile of L trunk=140 µm (LPh=70, L tail= 588, mm) also from the Coral Reserve (Eilat), Israel. The tail may reach 4 × the length of the trunk, but is sometimes broken to lengths even shorter than that of the trunk.

###### Etymology:

 Toxostylis (Greek: *toxo* + *stylos* = meaning ‘bow shaped column’) referring to the bowed shape of the reproductive stylet in the caudal organ.

###### Taxonomic affinities:

 *Urodasys toxostylis* sp. n. is the only species in the genus with an bluntly ovate head, pestle organs, a tail up to 4 times the length of the trunk, and a PhJIn of U32–U46, which also has TbA 7 per side in transverse rows and longitudinal columns; a TbL formula of 4=0,0/4 (0 along the fore and 0 along the rear pharynx and 4 along the intestine); a TbVL formula of 4=2,0/2; a TbD formula of 5=1,0/4; and TbP=10 or more per side, depending on the tail length, with TbV absent. There are several stylet-bearing species that are morphologically close to *Urodasys toxostylis* sp. n., all western Atlantic. *Urodasys cornustylis* Schoepfer-Sterrer, 1974 differs from *Urodasys toxostylis* sp. n. in having a stylet with an asymmetrical opening; *Urodasys nodostylis* Schoepfer-Sterrer, 1974 differs in having a stylet with a sharp nearly perpendicular angle at its base; *Urodasys calicostylis* Schoepfer-Sterrer, 1974 differs in having a stylet that lacks a bulb and narrows too quickly from its conical end-piece; and *Urodasys remostylis* Schoepfer-Sterrer, 1974 has a stylet that bears a false bulb, though the outer surface does not really indent.

### Family THAUMASTODERMATIDAE Remane, 1927 Genus Tetranchyroderma Remane, 1926

#### Tetranchyroderma corallium sp. n.

urn:lsid:zoobank.org:act:B550FEA5-7A3D-4329-9239-43DADD78A7C1

http://species-id.net/wiki/Tetranchyroderma_corallium

[Tet corl]

Figure 13

Tetranchyroderma  EgyF Hummon (2009) [E Med & Red Seas Database]

##### Diagnosis:

 Adult Lt 332 µm; PhJIn at U29. Body short, robust; head end truncated, without pestle organs, tentacles or lobes; lacking any neck; trunk broadening through out the pharyngeal region, then even more along the fore-gut, before narrowing gradually to the caudal base; cirrata 6 per side of nearly similar lengths, dorsolateral at U10, U30, U45, U62, U79 and U96; caudal pedicles medium, borne on fleshy lobes, with a broad concave margin separating the two lobes, incising medially to U94. Glands 6 per side (3–8 µm diameter) in lateral columns at U15–U79. Epidermis covered with pentancres twice as long as wide, with the center tine 20% longer than the others, though ancres are smaller fore and aft; ancres occur in 55–60 rows of 15–17 ancres each, extending onto the rear of the oral hood and onto the caudal lobes. TbA 7 per side forming 3 rows of 2, 3 and 2 tubes, all projecting obliquely forward, tubes inserting directly on the postoral body surface at U05–U07; TbVL 18 per side, 1 at U08, a group of 11 at U25–U71, 1 at U80, and a group of 5 at U86–U95; TbV 3 per side, 1 at U65 and 2 at U72; TbDL 4 per side (L 17–19 µm) at at U13, U47, U64 and U80; TbL/D *per se* absent; TbP 3 per side on the caudal pedicles, forming the fused ‘two fingers and a thumb’ typical of the family, supplemented by the last of the dorsal cirrata, with 5 additional tubes in the space between the peduncles. Locomotor ciliature: a single field that covers the entire ventral surface from TbA to the anus. Mouth subterminal, as broad as the fore end of the body, lightly cuticularized buccal cavity, pharynx with inconspicuous basal pores; intestine narrows fore to aft, anus ventral at U91. Hermaphroditic; testis on left side as seen from below; vas deferens opens in front of the anus; developing ovum occurs above the hind-gut; caudal organ ovoid and thick-walled; frontal organ spherical, hyaline, bearing active sperm, partly embedded in the rear of the ovum.

##### Description:

**Figure 13.**
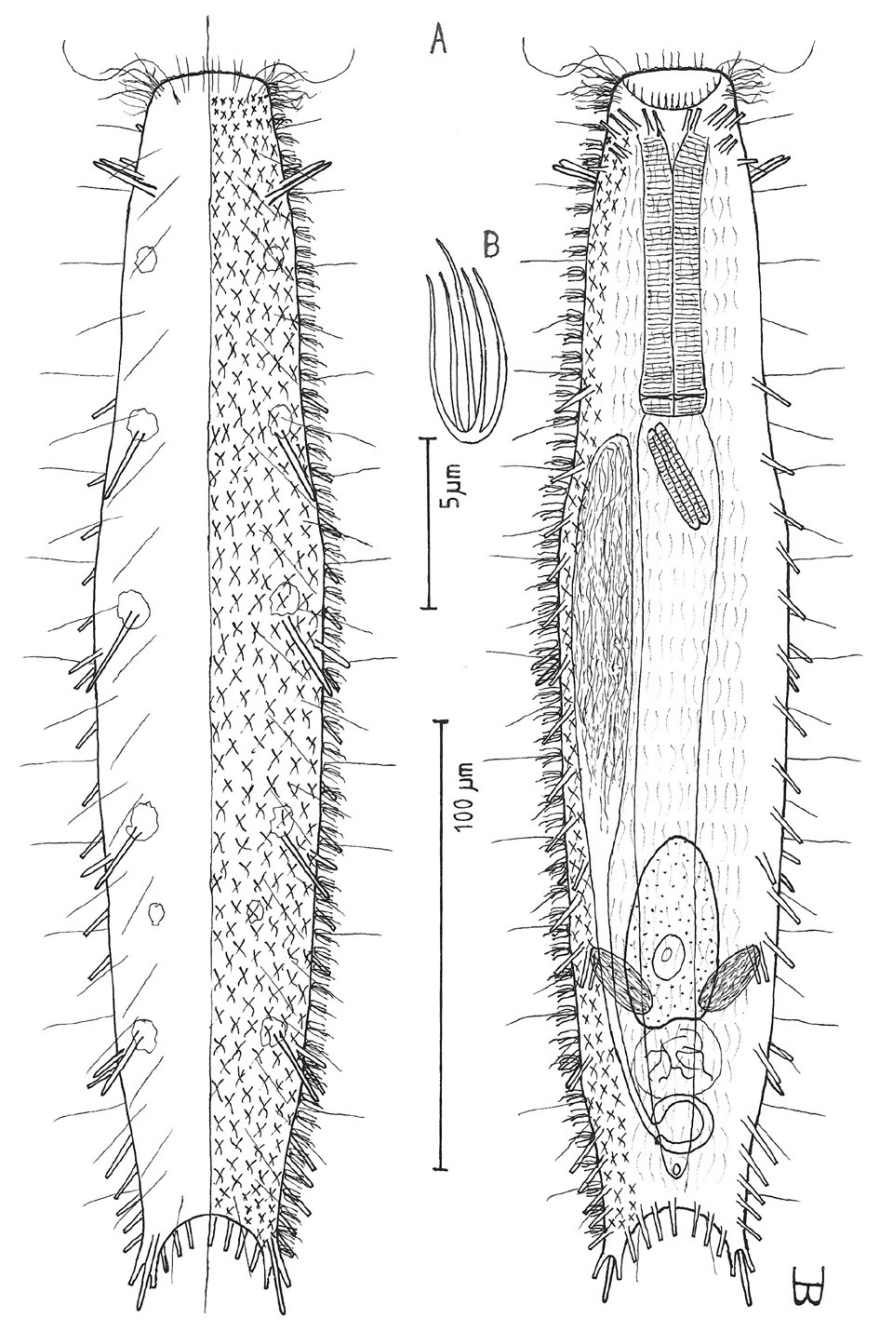
*Tetranchyroderma corallium* sp. n. **A** dorsal and ventral views of a mature adult (Lt=280, LPh=80 µm) from Middle Garden, S. Sinai, Egypt; dorsal with pentancrous surface (over half of the body), dorsal and lateral body cilia, and cirrata; ventral with digestive and reproductive tracts, adhesive tubes, and the locomotor ciliary band **B** dorsal pentancre, with a separate scale bar.

 Adult Lt 280–332 µm; LPh 85–80 µm to PhJIn at U30–U29 (Fig. 13). Body short, robust, ventrally flattened, dorsally vaulted; head end truncated, without pestle organs, tentacles or lobes; lacking any neck, broadening through out the pharyngeal region, then even more along the fore-gut, before narrowing gently to the caudal base; cirrata 6 per side of nearly similar lengths (L 16–22 µm), dorsolateral at U10, U30, U45, U62, U79 and U96, the last one homologous with the cirratum that is so often associated with the pedicles; caudal pedicles medium (L 17 µm), borne on fleshy lobes, with a broad concave margin separating the two lobes, incising medially to U94. Widths at mouth /rear pharynx /mid-gut /caudal base, and locations along the length of the body are as follows: 26 /49 /53 /32 µm at U01 /U29 /U48 /U96, respectively. Glands 6 per side (3–8 µm diameter) scattered in lateral columns at U15–U79.

*Cuticular armature*: Epidermis armored with pentancres (L 5, W 2 µm), much taller than wide, the central tine 20% longer (L 6 µm) than the other four (Fig. 13 B); ancres of similar size over much of the body, but are smaller fore and aft; ancres cover dorsal and lateral surfaces in some 55–60 rows of 15–17 ancres each, extending onto the rear of the oral hood and onto the caudal lobes.

*Adhesive tubes*: TbA 7 per side (L 4–6 µm), forming three rows of 2 (medial), 3 (lateral) and 2 (smaller, behind the lateral) tubes, all projecting obliquely forward and all inserting directly on the postoral body surface at U05–U07; TbVL 18 per side (L 4–10 µm), 1 at U08, inserting just behind the TbA, a group of 11 at U25–U71, 1 at U80, and a group of 5 at U86–U95, the first tube being shorter than the others; TbV 3 per side (L 7–9 µm), 1 at U65 and a row of 2 at U72; TbDL 4 per side (L 7–10 µm) at U13, U47, U64 and U80; TbL/D *per se* are absent; TbP 3 per side on the caudal pedicles, forming the fused ‘two fingers and a thumb’ typical of the family, (L terminal tubes 4–5 µm, L tube on the inner margin 6 µm), supplemented by the last of the dorsal cirrata, with 5 additional tubes (L 8–9 µm) in the space between the peduncles.

*Ciliation*: Short sensory cilia occur around the ventral oral opening (L 4 µm), with a number on the oral hood (L 7–8 µm), 1 longer per side (L 36 µm) being quite active, as well as numerous cilia laterally (L 5–12 µm); other cilia (L 12–20 µm) occur regularly along the lateral, dorsolateral and dorsal body surfaces, numbering 12–13 each. Ventral locomotor ciliature forms a single field of transverse rows from TbA to anus, lying between the TbVL columns; individual cilia are 6–8 µm in length.

*Digestive tract*: Mouth subterminal, as broad as the fore end of the body (23 µm width); oral hood extends from U00 to U04; buccal cavity is lightly cuticularized; pharynx has inconspicuous basal pharyngeal pores; intestine narrows gradually front to rear; anus is ventral at U91.

*Reproductive tract*: Hermaphroditic, testis on right side as seen from above (left side as seen from below); vas deferens appears to open into the caudal organ in front of the anus; the developing ovum (up to 49 × 22 µm) occurs above the hind-gut; caudal organ ovoid (14 µm outer diameter) is thick-walled, except where the vas deferens enters; frontal organ spherical and hyaline, bearing active sperm, partly embedded in the rear of the ovum.

##### Ecology:

 Sparse in frequency of occurrence (fewer than 10% of samples), rare to scarce in abundance (fewer than 1% to 5% of a sample); *littoral* in medium fine to medium, medium sorted coralline sand at low water neap to low water spring, 0–10 cm depth; *sublittoral* in fine to medium-fine, well to medium sorted coralline sand at 1.5–3 m water depth (sometimes in very fine to very coarse, very poorly sorted coralline sand at 14 m water depth, between coral platforms).

##### Geographical distribution:

 **RED SEA:** *EGYPT* {Sharm el-Arab Inside, Wadi ’Araba [video], Marsa Bareika N, ^Middle Garden (27°54'N, 34°21'E) [2-videos]}.

##### Remarks:

 There are three sequences of *Tetranchyroderma corallium* sp. n., all are from the upper Red Sea of Egypt. These are available as MPEG 2 (and MPEG 1) from Hummon (2009) #1519 a mature adult of Lt=332 µm (LPh=110 µm) from the Wadi ’Araba, Egypt; #1518 a mature **Lectotype** adult of Lt=280 µm (LPh=85 µm), collected in July 1994 from Middle Garden, off Ras Mohamed National Park, S. Sinai, Egypt; and #1516 a subadult of Lt=156 µm (LPh=79 µm) also from Middle Garden.

##### Etymology:

 The species is named with reference to the coralline-calcareous sediment in which it was always found.

##### Taxonomic affinities:

 *Tetranchyroderma corallium* sp. n. is the only small species in the genus without pestle organs, tentacles or lobes, a PhJIn at U29–U33, 6 cirrata of similar size and spacing per side, and pentancres twice as long as wide, with a more elongate central tine, which also has TbA 7 per side in three rows of 2, 3 and 2 tubes; TbVL 17 per side, solitary tubes at U08 and U80, and two groups of 11 at U25–U71 and 5 at U86–U95; TbV 3 per side, 1 at U65 and 2 at U72; TbDL 4 per side at U13, U47, U64 and U80; TbP 3 per side as ‘two fingers and a thumb’ on small pedicles supplemented by the last of the dorsal cirrata, and 5 additional tubes in the space between pedicles. There are only two pentancrous species, whose central tine is longer than the other tines: *Tetranchyroderma polyacanthus* (Remane, 1926) and *Tetranchyroderma tanymesathrum* Hummon, Todaro, Balsamo & Tongiorgi, 1996, neither of which have the TbD or TbV that are present in *Tetranchyroderma corallium* sp. n.

#### Tetranchyroderma rhopalotum sp. n.

urn:lsid:zoobank.org:act:63A062C4-0068-4B9E-B9FB-1BC20789AF90

http://species-id.net/wiki/Tetranchyroderma_rhopalotum

[Tet rplo]

Figure 14

Tetranchyroderma  EgyC Hummon (2009) [E Med & Red Seas Database]

##### Diagnosis:

 Adult Lt 344 µm; PhJIn at U30. Head end bluntly rounded, bearing club-shaped, ear-like pestle organs at U04; body narrows along the hind-pharynx; trunk broadens along the mid-gut, narrowing gently to the caudal base; caudal pedicles medium, with a sharply concave margin, indenting medially to U94. Glands 37 per side in medial and lateral columns. Epidermis covered with tetrancres of similar size, but smaller fore and aft; ancres occur in 36–38 rows of 13–15 ancres each, excluding the oral hood, but covering the caudal lobes. TbA 5 per side forming a transverse arc, tubes inserting directly on the postoral body surface; TbVL 12 per side, 1 along the fore pharynx, behind the TbA, 4 along the fore-gut, and 7 closely spaced from anus onto the caudal lobes; TbV 3 per side in a transverse row at U87; TbDL 2 per side at U51 (L 26 µm) and U90 (L 17 µm); TbP 3 per side on the caudal pedicles, forming the fused ‘two fingers and a thumb’ typical of the family, without supplemental cirrata-like structure projecting from between the ‘fingers’ or additional tubes in the space between the peduncles. Locomotor ciliature: a single field covers the entire ventral surface from TbA to anus, with a narrower tract continuing beneath the caudum. Mouth subterminal, as broad as the fore end of the body, buccal cavity lightly cuticularized; pharynx narrows to inconspicuous basal pharyngeal pores; intestine narrows fore to aft, anus ventral at U87. Hermaphroditic; testis on left side as seen from below; vas deferens opens near the anus; developing ovum occurs above the midgut, with oocytes bilaterally to the rear; caudal organ small, ovoid and thick-walled, with an interior of refractive material and a central canal; frontal organ broadly oval and hyaline, bearing active sperm.

##### Description:

**Figure 14.**
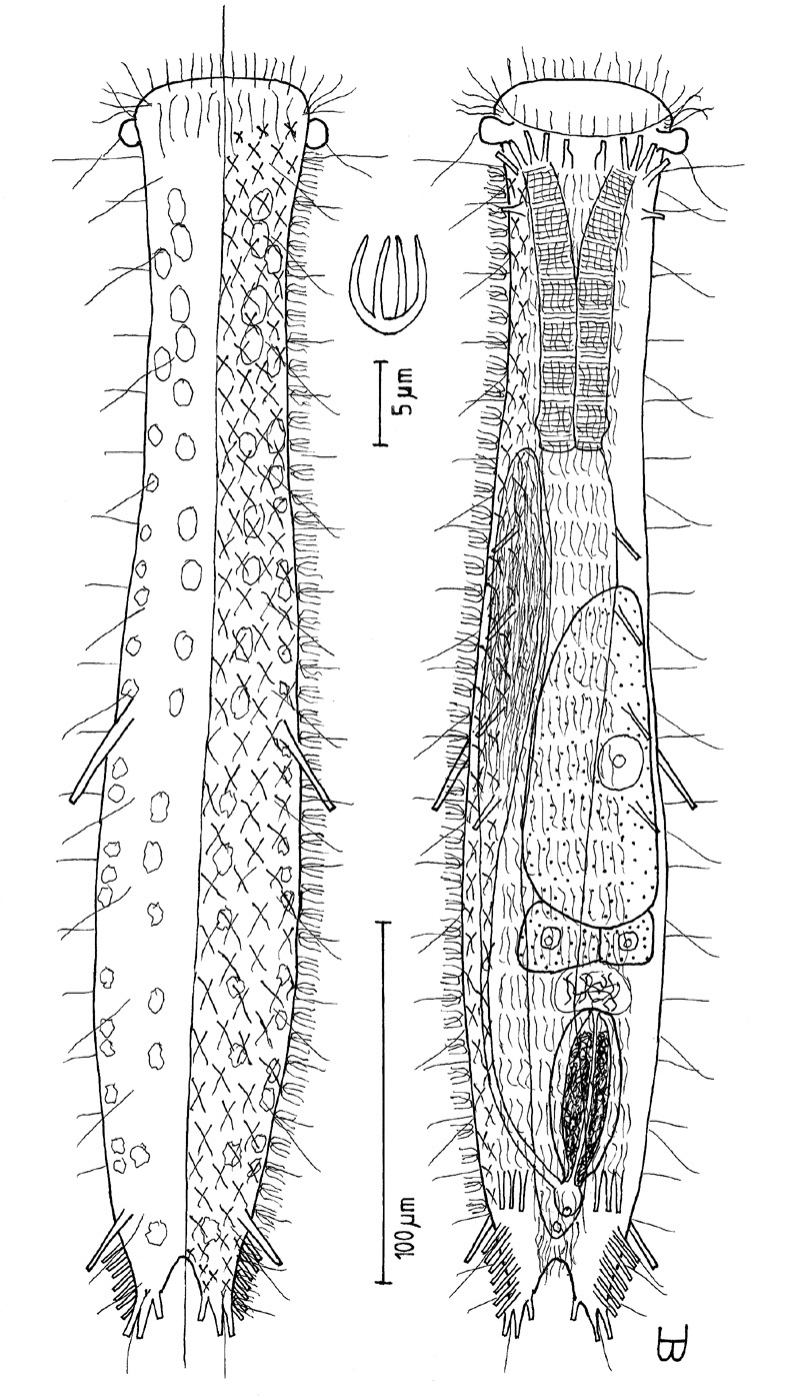
*Tetranchyroderma rhopalotum* sp. n. **A** dorsal and ventral views of a mature adult (Lt=344, LPh=102 µm) from the Giftun Village Spit Outside, near Hurghada, Egypt; dorsal with tetrancrous surface (over half of the body), dorsal and lateral body cilia, and dorsal adhesive tubes; ventral with digestive and reproductive tracts, other adhesive tubes, and the locomotor ciliary band **B** dorsal tetrancre, with a separate scale bar.

 Adult Lt 271–344 µm; LPh 102 µm to PhJIn at U39–U30 (Fig. 14). Body length medium, ventrally flattened, dorsally vaulted; head end bluntly rounded, bearing club-shaped, ear-like pestle organs at U04; body narrows along the hind-pharyx; trunk broadens along the mid-gut, narrowing gently to the caudal base; caudal pedicles of medium length (L 22 µm) borne on lobes, with a sharply concave margin separating the two lobes, indenting medially to U94. Widths at pestles /neck /mid-gut /caudal base, and locations along the length of the body are as follows: 48 /38 /56 /26 µm at U04 /U19 /U64 /U96, respectively. Glands 37 per side, 20 (2 µm diameter to 9 × 5 µm) scattered in lateral columns at U13–U87 and 16–17 (5 µm diameter to 9 × 5 µm) scattered in medial columns at U11–U92.

*Cuticular armature*: Epidermis armored with tetrancres (L 7 W 5 µm) that are of much the same size over much of the body (Fig. 14 B), though somewhat smaller fore and aft; ancres cover dorsal and lateral surfaces in some 36–38 rows of 13–15 ancres each, being absent from the oral hood, but extending the length of the caudal lobes.

*Adhesive tubes*: TbA 5 per side (L 9 µm), forming a transverse arc, the medial-most differing in shape from the others, all tubes inserting directly on the postoral body surface at U07–U08, radiating from forward to obliquely outward. TbVL 12 per side, 1 in the fore pharyngeal region (L 6 µm) at U10 inserting just behind the TbA, 4 of similar size and spacing along the fore-gut (L 10 µm) at U36–U58, and 7 located at and behind the anus (L 10 µm) closely spaced at U91–U96, the last 3 inserting beneath the caudal lobe; TbV 3 per side (L 10 µm) in a transverse row at U87; TbDL 2 per side at U51 (L 26 µm) and U90 (L 17 µm); TbP 3 per side on the caudal pedicles, forming the fused ‘two fingers and a thumb’ typical of the family, (L terminal tubes 8 µm, L tube on the inner margin 6 µm), not supplemented by a blind cirrata-like structure projecting dorsoposteriorly from between the ‘fingers,’ and with no additional tubes in the space between the peduncles.

*Ciliation*: Many short sensory cilia surround the oral opening (L 6–9 µm), with a number along the fore end of the oral hood (L 10–18 µm) and several laterally (L 12–15 µm) before the pestle organs; other cilia occur regularly along the lateral (L 10–40 µm) and dorsolateral (L 22–40 µm) body surfaces, numbering 12 and 18 per side, respectively. Ventral locomotor ciliature forms a single field of transverse rows from TbA to anus, lying between the TbVL columns, the field narrowing from anus to caudal base; individual cilia are 8–10 µm in length.

*Digestive tract*: Mouth subterminal, as broad as the fore end of the body (32 µm width); oral hood extends from U00 to U03; buccal cavity is lightly cuticularized; pharynx has inconspicuous basal pharyngeal pores; intestine narrows gradually front to rear; anus is ventral at U87.

*Reproductive tract*: Hermaphroditic, testis on right side as seen from above (left side as seen from below); vas deferens appears to open into the caudal organ, just before the anus; The largest developing ovum (up to 94 × 37 µm) occurs above the foregut, with ovules bilaterally to the rear; caudal organ large, longitudinally ovoid and thick-walled (47 × 19 µm), with an interior of refractive material and a central canal; frontal organ transversally ovoid and hyaline, often bears active sperm.

##### Ecology:

 Occasional in frequency of occurrence (10–30% of samples), scarce to prevalent in abundance (3% to greater than 30% of a sample, occasionally a co-dominant **[cdom]**); *littoral* in fine to very coarse, medium to poorly sorted clean silicious or carbonate sand at low water neap to low water spring, 0–15 cm depth; *sublittoral* in very fine to medium, well to medium sorted sand at 1–3 m water depth (sometimes in very fine-very coarse, poorly to very poorly sorted coralline sand at the edge of or amid a depression in a coral platform at 1 m water depth).

##### Geographical distribution:

 **RED SEA:** *EGYPT* {23km S Ein Sukhna, Sharm el-Arab Outside, Wadi ’Araba [video], Daghashland, Giftun Island SE [2-videos], Giftun Island SS, ^Giftun Village Spit Outside **[cdom]** (27°10'N, 33°49'E) [4-videos], Hammam Pharaon, Nabq S [video], Ras Nasrani}.

##### Remarks:

 There are eight video sequences of *Tetranchyroderma rhopalotum* sp. n., all are from the upper Red Sea in Egypt. Five of these are available as MPEG 2 (and MPEG 1) from Hummon (2009): #1494 a mature **Lectotype** adult of Lt=344 µm (LPh=118 µm), collected in June 1994 from the Giftun Village Spit O, near Hurghada; #1500 a mature adult of Lt=303 µm (LPh=109 µm) from Nabq, S. Sinai; #1501 a mature adult of Lt=271 µm (LPh=107 µm) from Wadi ‘Araba, Gulf of Suez; #1497 a subadult of Lt=186 µm (LPh=79 µm) also from the Giftun Village Spit O; and the last, #1499 a juvenile of Lt=123 µm (LPh=57 µm) from Giftun Island SE.

##### Etymology:

 Rhopalotum (Greek: *rhopalon* + *otus* = meaning ‘club’-like ‘ear’) referring to its club-shaped, ear-like pestle organs.

##### Taxonomic affinities:

 *Tetranchyroderma rhopalotum* sp. n. is the only medium sized species in the genus with club-shaped, ear-like pestle organs, a PhJIn at U39–U30, and tetrancres, which also has TbA 5 per side; TbVL 12 per side, 1 behind the TbA, 4 along the fore half of the intestine, and a set of 7 near the caudum; TbV 3 in a row on each side at U87; TbDL 2 at U51 (longer) and U90 (shorter); TbP 3 per side as ‘two fingers and a thumb’ on medium pedicles without a cirrata-like structure inserting between the ‘fingers’ or additional tubes in the space between pedicles. Only two other described species that bear only tetrancres, also have lateral cephalic tentacles and TbV: *Tetranchyroderma sanctaecaterinae* Todaro, Tongiorgi & Balsamo, 1992 and *Tetranchyroderma schizocirratum* Chang, Kubota & Shirayama, 2002, the former has its TbV in columns and lacks TbD, while the latter, like *Tetranchyroderma rhopalotum* sp. n., has its TbV in rows and has TbD; *Tetranchyroderma schizocirratum* differs from *Tetranchyroderma rhopalotum* sp. n. in having asymmetrical rather than symmetrical pestle organs and three dorsal cirrata per side, whereas dorsal cirrata are absent in *Tetranchyroderma rhopalotum* sp. n.

#### Tetranchyroderma sinaiensis sp. n.

urn:lsid:zoobank.org:act:D93DAC26-9140-40C9-A797-D8507B3F6877

http://species-id.net/wiki/Tetranchyroderma_sinaiensis

[Tet snai]

Figure 15

Tetranchyroderma  EgyD Hummon (2009) [E Med & Red Seas Database]

##### Diagnosis:

 Adult Lt 423 µm; PhJIn at U34. Body of medium length, narrow; head end rounded, without pestle organs, tentacles or lobes; neck indistinct along the rear pharynx, trunk broadening slightly in the mid-body, before narrowing gently to the caudal base; caudal pedicles short, borne on short lobes, with a wide concave margin separating the two lobes, incising medially to U97. Glands 8 per side, small, scattered in lateral columns. Epidermis covered with tetrancres of similar size, slightly smaller fore and aft; ancres occur in 45–50 rows of 9–10 ancres each, excluding the entire oral hood, but extending onto the base of the caudal lobes. TbA 7 per side forming arcs radiating from forward to outward, tubes inserting directly on the postoral body surface at U03–U05; TbVL 43 per side, 1 at U06, a group of 36 at U25–U80, 1 at U89, and a group of 5 at U93–U97; TbV 5 per side in a transverse row at U85; TbDL 9 per side, of two sizes, 7 (short) subequally spaced at U10–U90, and 2 (long) at U86 and U93; TbL/D *per se* absent; TbP 5 per side on the caudal pedicles, forming the fused ‘two fingers and a thumb’ typical of the family, supplemented by a cirrata-like structure projecting from between the ‘fingers,’ with 5 additional tubes in the space between the peduncles. Locomotor ciliature: a single field that covers the entire ventral surface at U04–U96. Mouth subterminal, as broad as the fore end of the body; buccal cavity lightly cuticularized; pharynx, broad throughout, has inconspicuous basal pores; intestine narrows fore to aft, anus is ventral at U94. Hermaphroditic; testis on left side as seen from below; vas deferens appears to open in front of the anus; developing ovum occurs above the hindgut; caudal organ is longitudinally ovoid and thick-walled, with an interior of refractive material and a central canal in the fore half; frontal organ is ovoid, hyaline and bi-layered, bearing active sperm, and partly embedded in the rear of the ovum.

##### Description:

**Figure 15.**
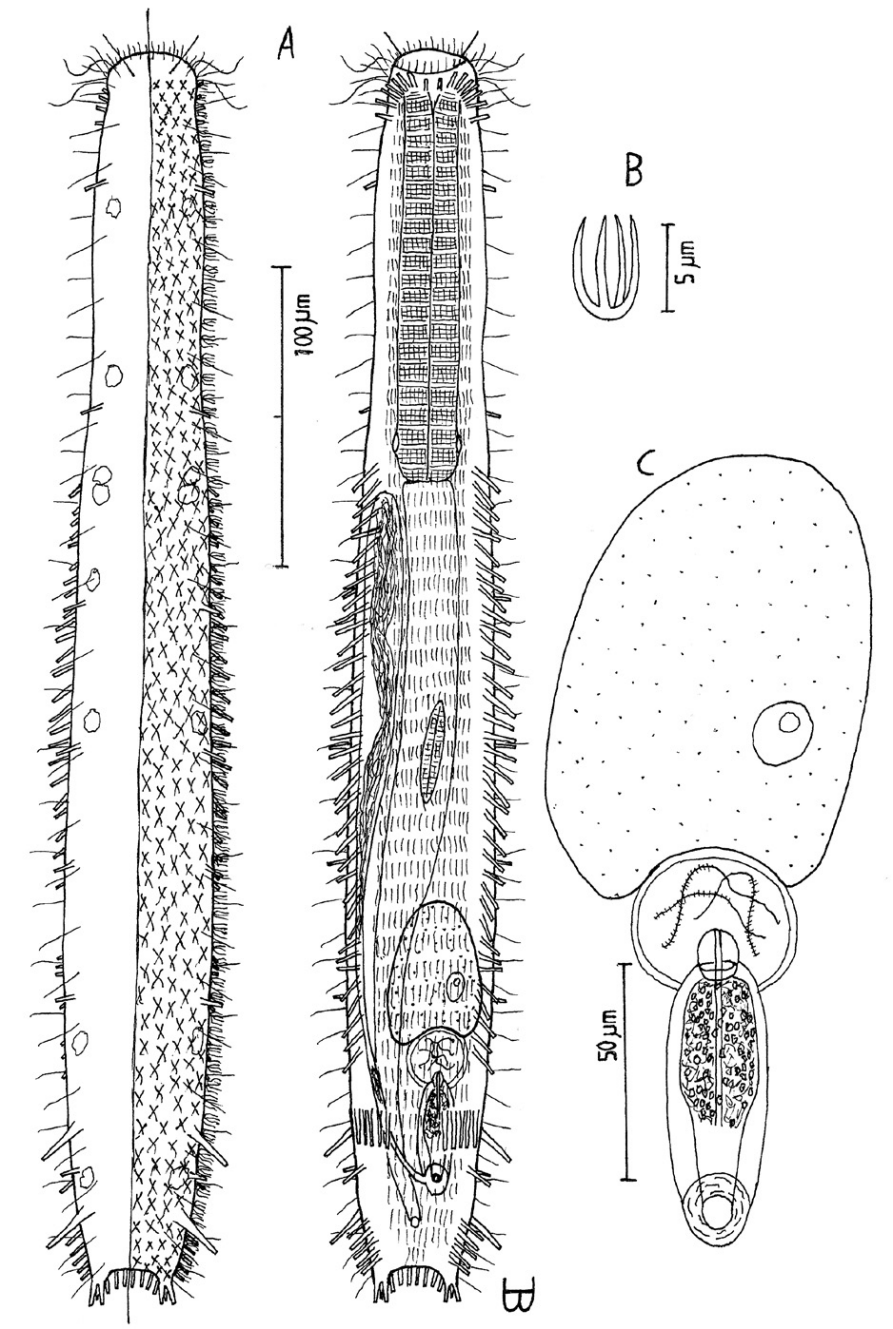
*Tetranchyroderma sinaiensis* sp. n. **A** dorsal and ventral views of a mature adult (Lt=423, LPh=146 µm) from the Na’ama Bay, S. Sinai, Egypt; dorsal with tetrancrous surface (over half of the body), dorsal and lateral body cilia, and dorsolateral adhesive tubes; ventral with digestive and reproductive tracts, oyher adhesive tubes, and the locomotor ciliary band **B** dorsal tetrancre **C** caudal organ, frontal organ and ovum; B. and C. with separate scale bars.

 Adult Lt 423 µm (others Lt 241–410); LPh 146 µm (others LPh 95–141) to PhJIn at U34 (others PhJIn at U39–U34) (Fig. 15). Body of medium length, narrow, ventrally flattened, dorsally vaulted; head end rounded, without pestle organs, tentacles or lobes; neck indistinct along the rear pharynx, trunk broadening slightly in the mid-body, before narrowing gently to the caudal base; caudal pedicles short (L 11 µm) borne on short lobes, with a wide concave margin separating the two lobes, incising medially to U97. Widths at mouth /rear pharynx /mid-gut /caudal base, and locations along the length of the body are as follows: 21 /36 /49 /27 µm at U02 /U25 /U61 /U97, respectively. Glands 8 per side (4–6 µm diameter) scattered in lateral columns at U08–U90.

*Cuticular armature*: Epidermis armored with tetrancres, taller than wide (L 6, W 3 µm), of much the same size over much of the body, slightly smaller fore and aft; ancres cover dorsal surface in some 45–50 rows of 9–10 ancres each, being absent from the oral hood, but extending onto the base of the caudal lobes.

*Adhesive tubes*: TbA 7 per side (L 3–5 µm), forming arcs radiating from directly forward to obliquely outward, the medial-most being smaller than the other 6 and set slightly to the rear of the arc, all tubes inserting directly on the postoral body surface at U03–U05. TbVL 43 per side, 1 at U06 (L 8 µm), just behind the TbA, a group of 36 at U25–U80 (L 10–12 µm), 1 at U89 (L 10 µm), and a group of 5 at U93–U97 (L 10–12 µm); TbV 5 per side (L 11–12 µm) in a transverse row at U85; TbDL 9 per side, of two sizes, 7 (L 6–8 µm) subequally spaced at U10, U28, U44, U55, U65, U75 and U90, and 2 (L 17–19 µm) at U86 and U93; TbL/D *per se* are absent; TbP 3 per side on the caudal pedicles, forming the fused ‘two fingers and a thumb’ typical of the family, (L terminal tubes 7 µm, L tube on the inner margin 4 µm), supplemented by a blind cirrata-like structure projecting dorsoposteriorly from between the ‘fingers,’ with 5 additional tubes (L 5 µm) in the space between the peduncles 3 on one side and 2 on the other.

*Ciliation*: Short sensory cilia surround the oral opening (L 2–4 µm), with a number on the oral hood and several laterally (L 15–24 µm), the longer ones being vibratile; other cilia occur regularly along the lateral (L 8–11 µm) and dorsolateral (L 14–17 µm) body surfaces, numbering 23 each. Ventral locomotor ciliature forms a single field of transverse rows from TbA to anus, lying between the TbVL columns, the field narrowing from anus to caudal base; individual cilia are 6–8 µm in length.

*Digestive tract*: Mouth subterminal, as broad as the fore end of the body (15 µm width); oral hood extends from U00 to U02; buccal cavity lightly cuticularized; pharynx, broad throughout, has inconspicuous basal pores; intestine narrows gradually front to rear and bends around the reproductive organs in the hind-gut region; anus is ventral at U94.

*Reproductive tract*: Hermaphroditic, testis on right side as seen from above (left side as seen from below); vas deferens opens in front of the anus; developing ovum (up to 48 × 32 µm) occurs above the hindgut; caudal organ, large, longitudinally ovoid and thick-walled (43 × 12 µm), has an anterior protuberance, a concentric circular pore in the rear, an interior of refractive material, and a central canal in the fore half; frontal organ, ovoid, hyaline and bi-layered, bears active sperm, and is partly embedded in the rear of the ovum.

##### Ecology:

 Common in frequency of occurrence (30–60% of samples), scarce to prevalent in abundance (3% to greater than 30% of a sample, occasionally a co- **[cdom]** or dominant **[dom]**); *sublittoral* in very fine to medium, well to medium sorted sand at 0.5–10 m water depth (sometimes in very fine-very to coarse, poorly to very poorly sorted sand at 2–15 m water depth, amid fringing corals or between coral platforms, of both healthy and unhealthy corals, or at 1.5 m water depth at the base of a seagrass bed).

##### Geographical distribution:

 **RED SEA:** *EGYPT* {Abu Ramada 1 [video], Abu Ramada 2 **[dom]**, Abu Ramada 3, Marsa Bareika N **[dom]**, Daghashland [video], Far Garden [2-videos], Giftun Island SE [video], Main Beach Ras Mohamed NP **[cdom]**, Moon Beach, Moon Valley, ^Na’ama Bay S (27°53'N, 34°19'E) [3-videos], Nabq S [video], Ras Nasrani, Ras Qanti [video], Tareef el-Reeh S **[dom]}**; *ISRAEL:* {Coral Beach M2, M3, M4 & M5, Princess Hotel [video], Snuba DS [video]}.

##### Remarks:

 There are 12 video sequences of *Tetranchyroderma sinaiensis* sp. n., all from the upper Red Sea in Egypt and Israel. Five of these are available as MPEG 2 (and MPEG 1) from Hummon (2009): #1505 a mature **Lectotype** adult of Lt=423 µm (LPh=146 µm), collected in July 1994 from Na’ama Bay, S. Sinai, Egypt; #1504 a mature adult of Lt=410 µm (LPh=141 µm) from Giftun Island SE, near Hurghada, Egypt; #1508 a mature adult of Lt=371 µm (LPh=110 µm) from Ras Qanti, S. Sinai, Egypt; #1506 a mature adult of Lt=241 µm (LPh=95 µm) also from Na’ama Bay; and #1509 a juvenile of Lt=154 µm (LPh=73 µm) from Far Garden, S. Sinai, Egypt.

##### Etymology:

 Sinaiensis (pronounced ‘sinaiënsis’ with a dieresis over the ‘e’ to indicate that it is to be pronounced separately from the diphthong ‘ai’) is named after the geographical region, the Sinai Peninsula, in which it was first found.

##### Taxonomic affinities:

 *Tetranchyroderma sinaiensis* sp. n. is the only species of medium length in the genus without pestle organs, tentacles or lobes, a PhJIn at U39–U34, and tetrancres, which also has TbA 7 per side; TbVL 43 per side, two solitary tubes at U06 and U89, and two groups of 36 at U25–U80 and 5 at U93–U97; TbV 5 in a row on either side at U85, TbDL 9 per side, of two sizes, 7 short at U10–U90, and 2 long at U86 and U93, TbP 3 per side as ‘two fingers and a thumb’ on small pedicles with a blind cirrata-like structure inserting between the ‘fingers’ and 5 additional tubes in the space between pedicles, but lacking TbL/D *per se*. *Tetranchyroderma pachysomum* Hummon, Todaro & Tongiorgi, 1993 is the only other species in the genus that has tetrancres, TbD and TbV, but it is a short, squat animal, with differing numbers and arrangements of adhesive tubes than in *Tetranchyroderma sinaiensis* sp. n.

#### Tetranchyroderma xenodactylum sp. n.

urn:lsid:zoobank.org:act:63A40C57-1DE1-4EC1-A5E5-6A1DFE2FA0D0

http://species-id.net/wiki/Tetranchyroderma_xenodactylum

[Tet xndc]

Figure 16

Tetranchyroderma  EgyE Hummon (2009) [E Med & Red Seas Database]

##### Diagnosis:

 Adult being described Lt 246 µm; PhJIn at U35. Body short, robust; head end rounded, without pestle organs, tentacles or lobes; neck a slight narrowing at the PhJIn; trunk broadening in the mid-gut, before narrowing gently then quickly to the caudal base; cirrata dorsolateral, 3 per side; caudal pedicles medium, naked, with a narrow concave margin separating the pedicles, incising medially to U92. Glands 9 per side scattered in lateral columns at U24–U85. A strange sub-cylindrical finger-like structure occurs laterally at U34. Epidermis covered with curved pentancres three times as long as wide, slightly smaller fore and aft; ancres occur in 46 rows of 14–15 ancres each, on dorsal and lateral surfaces, extending onto the middle of the oral hood and onto the caudal base. TbA 4 per side, 1 medially and 3 laterally, all projecting forward or obliquely outward, tubes inserting directly on the postoral body surface at U09–U10; TbVL 11 per side, 1 along the fore half of the pharynx at U14, 7 along the intestine at U39–U75, and 3 at and behind the anus at U88–U92; TbV 3 per side in a transverse row at U80; TbL *per se*/TbD absent; TbP 3 per side on the caudal pedicles, forming the fused ‘two fingers and a thumb’ typical of the family, supplemented by the last of the dorsal cirrata, with 2 additional tubes in the space between the peduncles. Locomotor ciliature: a single field covers the ventral surface from TbA to the anus and behind. Mouth subterminal, as broad as the fore end of the body; buccal cavity lightly cuticularized; pharynx medium throughout, with inconspicuous basal pores; intestine narrows fore to aft, anus ventral at U88. Hermaphroditic; testis on left side as seen from below; vas deferens appears to open in front of the anus; developing ovum occurs above the hindgut; caudal organ spherical; frontal organ oblong, hyaline, partly embedded in the rear of the ovum.

##### Description:

**Figure 16.**
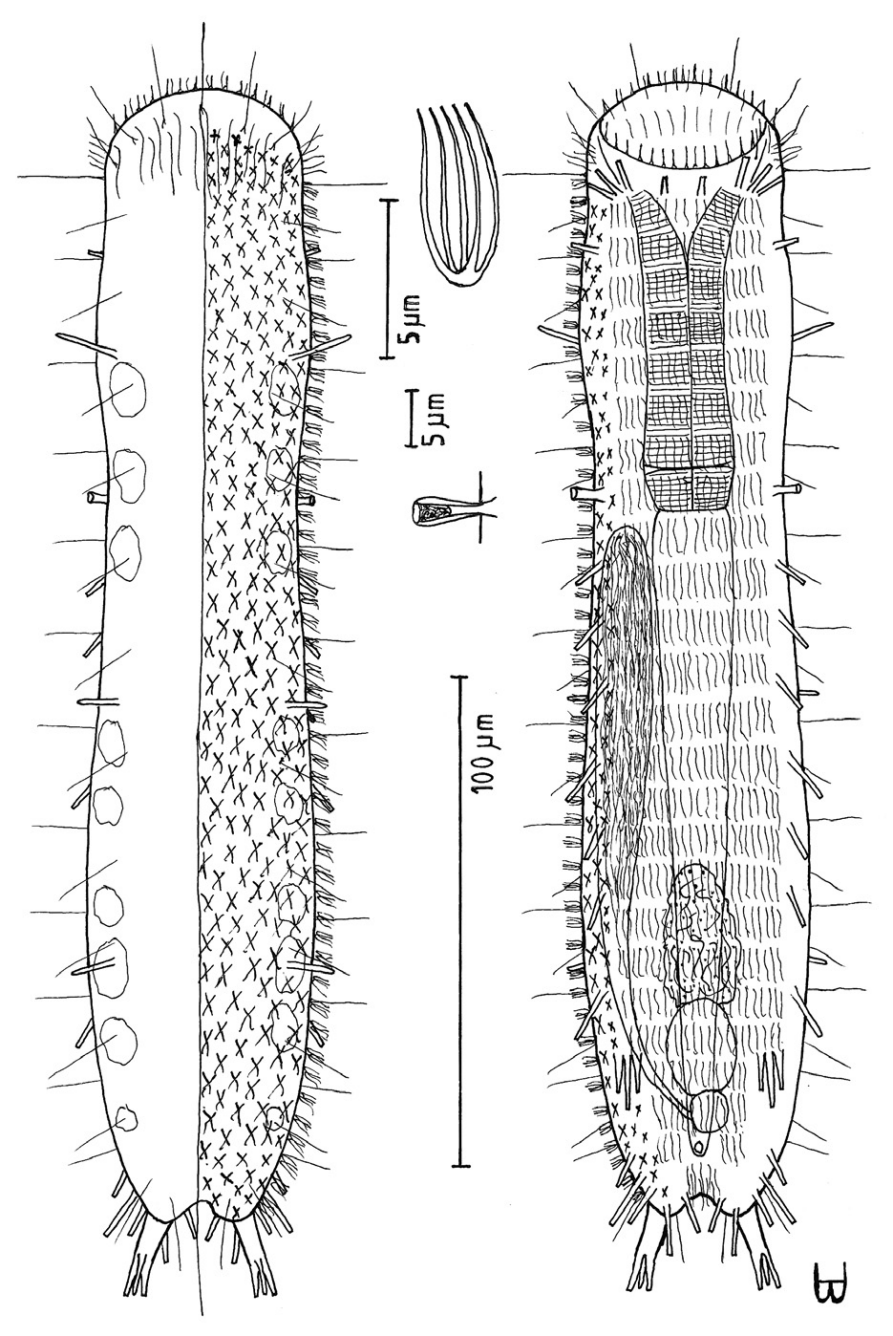
*Tetranchyroderma xenodactylum* sp. n. **A** dorsal and ventral views of a mature adult (Lt=246, LPh=87 µm) from the Nabq, S. Sinai, Egypt; dorsal with pentancrous surface (over half of the body), dorsal and lateral body cilia, and TbDL; ventral with digestive and reproductive tracts, adhesive tubes, and the locomotor ciliary band **B** dorsal pentancre **C** the strange finger-like structure that protrudes laterally at the PhJIn; B. and C. with separate scale bars.

 Adult being described Lt 246 µm (others 197–322); LPh 87 µm (others 79–128) to PhJIn at U35 (others to PhJIn at U40) (Fig. 16). Body short, robust, ventrally flattened, dorsally vaulted; head end rounded, without pestle organs, tentacles or lobes; neck a slight narrowing at the PhJIn, broadening along the mid-gut, before narrowing gently along the hind-gut and then quickly behind the anus to the caudal base; cirrata 4 per side (L 7–11 µm), dorsolateral at U22, U50, U72 and U96; caudal pedicles medium (L 13 µm), naked (without lobes), with a broad concave margin separating the two pedicles, incising medially to U92. Widths at mouth /mid-pharynx /PhJIn /mid-gut /caudal base, and locations along the length of the body are as follows: 41 /44 /39 /47 /22 µm at U06 /U19 /U34 /U66 /U93, respectively. A strange sub-cylindrical finger-like structure (L 7 µm) occurs at U34, being hollow but occluded at its outer end. Glands 9 per side (4 µm diameter to 6 × 9 µm) scattered in lateral columns at U24–U85.

*Cuticular armature*: Epidermis armored with slightly curved pentancres (L 6, W 2.5 µm), much taller than wide, all 5 tines of the same length, ancres of much the same size over most of the body, but slightly smaller fore and aft; ancres cover dorsal and lateral surfaces in some 46 rows of 14–15 ancres each, extending onto the middle of the oral hood and onto the caudal base.

*Adhesive tubes*: TbA 4 per side, 1 medially (L 4 µm) projecting forward and 3 laterally (L 6–8 µm), projecting obliquely to the side, all inserting directly on the postoral body surface at U09–U10. TbVL 11 per side, 1 (L 4 µm) along the fore half of the pharynx at U14, just behind the TbA, 7 (L 7–12 µm) along the intestine at U39–U75, and 3 (L 7–12 µm) at and behind the anus at U88–U92; TbV 3 per side (L 7, 11, 7 µm) in a transverse row at U80; TbL *per se* and TbD are absent; TbP 3 per side on the caudal pedicles, forming the fused ‘two fingers and a thumb’ typical of the many members of the family, (L terminal tubes 4–5 µm, L tube on the inner margin 9 µm), supplemented by the last of the dorsal cirrata, with 2 additional tubes (L 7–8 µm) in the space between the peduncles.

*Ciliation*: Short sensory cilia surround the oral opening (L 4 µm), with a number of also on the oral hood: 6 (L 14–17 µm) projecting directly or obliquely forward and 13–15 (L 7–14 µm) trailing to the rear; other cilia (L 10–18 µm) occur regularly along the lateral and dorsolateral body surfaces, numbering 12–13 each. Ventral locomotor ciliature forms a single field of transverse rows from TbA to behind the anus, lying between the TbVL columns; individual cilia are 5–6 µm in length.

*Digestive tract*: Mouth subterminal, as broad as the fore end of the body (34 µm width); oral hood extends from U00 to U07; buccal cavity lightly cuticularized; pharynx of medium breadth throughout, with inconspicuous basal pores; intestine narrows gradually front to rear; anus is ventral at U88.

*Reproductive tract*: Hermaphroditic, testis on right side as seen from above (left side as seen from below); vas deferens appears to open in front of the anus; developing ovum (up to 29 × 16 µm), appearing somewhat shrivled, occurs above the hindgut; caudal organ spherical (7 µm diameter); frontal organ oblong (20 × 14 µm) and hyaline, without active sperm, partly embedded in the rear of the ovum.

##### Ecology:

 Sparse in frequency of occurrence (fewer than 10% of samples), scarce to numerous in abundance (3% to 20% of a sample, sometimes a sub-dominant **[sdom]**); *littoral* in fine, well sorted sand at low water spring in a shallow tombolo, 0–5 cm depth; *sublittoral* in fine to medium-fine, well to medium-well sorted sand at 1–5 m water depth.

##### Geographical distribution:

 **RED SEA:** *EGYPT* {Marsa Bareika W, ^Nabq **[sdom]** (28°05'N, 34°35'E) [2-videos], Sharm el-Sheikh, Wadi ’Araba [video]}.

##### Remarks:

 There are three sequences of *Tetranchyroderma xenodactylum* sp. n., all from the upper Red Sea in Egypt. These three are available as MPEG 2 (and MPEG 1) from Hummon (2009): #1517 a mature adult of Lt=332 µm (LPh=128 µm) from the Wadi ’Araba, on west side of the Gulf of Suez, Egypt; #1514 a mature **Lectotype** adult of Lt=246 µm (LPh=91 µm), collected in July 1994 from Nabq, S. Sinai, Egypt; and #1515 a mature adult of Lt=197 µm (LPh=79 µm) also from Nabq.

##### Etymology:

 Xenodactylum (Greek: *xenos* + *daktylos*, meaning ‘strange finger’) is named after the strange finger-like appendage that protrudes laterally in all specimens from the rear of the pharyngeal region.

##### Taxonomic affinities:

 *Tetranchyroderma xenodactylum* sp. n. is the only small species in the genus without pestle organs, tentacles or lobes, a PhJIn at U40–U35 and slightly curved pentancres, with tines of similar size, which also has TbA 4 per side, 1 medial and 3 lateral, TbVL 11 per side, 1 along the fore half of the pharynx at U14, 7 along the intestine at U39–U75, and 3 at and behind the anus at U88–U92; TbV 3 per side in a transverse row at U80; TbP 3 per side on the caudal pedicles, with the fused ‘two fingers and a thumb’ typical of much of the family, supplemented by the last dorsal cirratum, with 2 additional tubes between the peduncles, TbL *per se* and TbD being absent. There is no other species in the genus, regardless of their ancres, that bears such a sub-cylindrical finger-like structure as is found at the base of the pharynx in *Tetranchyroderma xenodactylum* sp. n.

### Family TURBANELLIDAE Remane, 1927 Genus Paraturbanella Remane, 1927

#### Paraturbanella levantia sp. n.

urn:lsid:zoobank.org:act:85825B17-FC5A-4098-AEEF-DF85D4BE2C0F

http://species-id.net/wiki/Paraturbanella_levantia

[Ptb lvnt]

Figure 17

Paraturbanella  EgyA Hummon (2001, 2004, 2007, 2009) [E Med & Red Seas Database].
Paraturbanella levantina Todaro et al. (2003: Appx. I, listed as *nomen nudum* that was reported in a CD “Global Data Base for Marine Gastrotricha” Hummon, 2001*)

##### Diagnosis:

 Adult Lt 657 µm; PhJIn at U23. Body elongate, slender; mouth a narrow outwardly rolled protrusion, head with a band of circumcephalic cilia at U03 and prominent pestle organs at U04, but lacking lateral lobes; neck constriction lacking, body sides parallel over most of their length, thinning gradually to the caudal base; caudum is slightly cleft, incised from its tips to U97; medial cone usually absent. Glands inconspicuous, ca. 28 per side. TbA 8 per side, the shortest one inserting on the medial edge, occur on fleshy hands that insert at U11; TbL absent; TbD 7 per side at U27–U84 and TbV 14 per side at U29–U88, all in the intestinal region, of similar size and spacing; ‘dohrni’ [Seitenfüsschen] tubes 2 per side, posteriolaterally directed (L longer tube =20 µm, shorter =15 µm), inserting ventrolaterally just behind the fleshy hands at U12; TbP 8 per side, the outermost being the longest and thickest, the others being shorter, with none occurring on the lateral or leading edges of the lobes. Locomotor ciliature: 2 longitudinal bands run from the pestle organs back and join behind the level of the anus. Mouth terminal, breadth narrow; buccal cavity small, deep, vaseshaped; walls of medium cuticularization; basal pharyngeal pores large and conspicuous; intestine narrows gradually front to rear; anus is ventral at U93. Hermaphroditic, protandrous to simultaneous; paired testes extend rearward from just behind the PhJIn, their vasa deferentia recurving to the fore and exiting at about U31; small developing ova occur bilaterally in the mid-gut region; frontal and caudal organs not seen.

##### Description:

**Figure 17.**
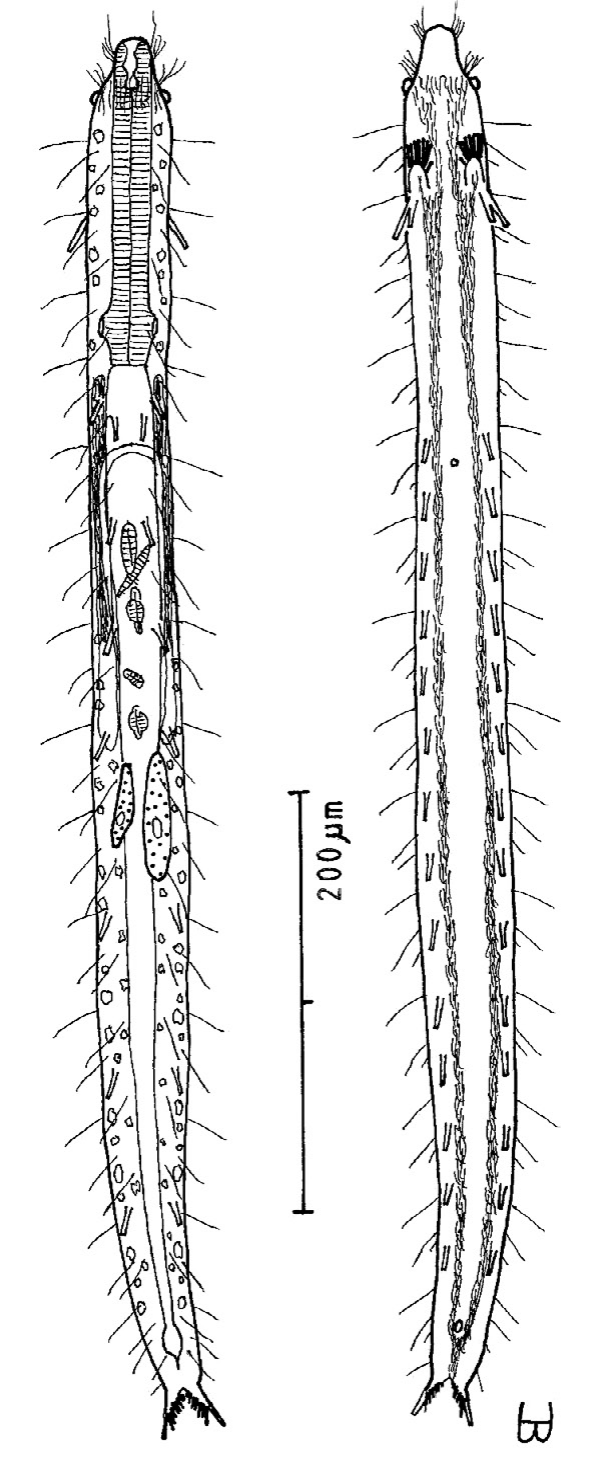
*Paraturbanella levantia* sp. n. dorsal and ventral views of a mature adult (Lt=657, LPh=163 µm) from Bir Mesud, Alexandria, Egypt; dorsal with pestle organs, pattern of glands, dorsal and lateral body cilia, digestive and reproductive tracts; ventral with adhesive tubes and locomotor ciliary bands.

 Adult Lt 635–657 µm; L to PhJIn 155–163 µm at U26–U23 (Fig. 17). Body elongate, slender; mouth a narrow outwardly rolled protrusion, head with a band of circumcephalic cilia at U03 and prominent pestle organs at U04, but lacking lateral lobes; neck constriction lacking, body sides parallel over most of their length, thinning gradually to the caudal base; caudum is slightly cleft, incised from its tips to U97; medial cone is usually absent. Widths at narrowed mouth /pestle organs /PhJIn /mid-trunk /furcal base, tips, and their locations along the body length are: 18 /31 /38 /44 /18, 30 µm at U02 /U04 /U23 /U58 /U97, U100. Epidermal glands ca. 28 per side, small (2–5 µm diameter), are distributed along the lateral body margins, but appear inconspicuous.

*Adhesive tubes*: TbA 8 per side (L 5–9 µm), the shortest one inserting on the medial most edge, occur on fleshy hands that insert at U11; TbL absent; TbD 7 per side (L 11–14 µm) from U27 to U84 and TbV 14 per side (L also 11–14 µm) from U29 to U88, all of similar size and spacing in the intestinal region; ‘dohrni’ [Seitenfüsschen] tubes 2 per side, posteriolaterally directed (L longer tube 20 µm, shorter 15 µm), inserting ventrolaterally immediately behind the fleshy hands at U12; TbP 8 per side, the outermost being the longest and thickest (L 14 µm), the others being shorter (L 3–9 µm), none occurring on the lateral or leading edges of the lobes.

*Ciliation*: Head protrusion has sensory hairs (L 11–30 µm) laterally and a circumcephalic band of cilia (L 14 µm) at U03; other sensory hairs (L 20–30 µm) occur on the trunk in lateral, dorsolateral and dorsal columns, with 14/14/18 per side. Ventral locomotor cilia (L=8 µm) flow from the circumcephalic band rearward in two longitudinal bands that trace the lateral body margins, joining again behind the level of the anus.

*Digestive tract*: Mouth terminal, narrow (8 µm diameter); buccal cavity small, deep, vaseshaped; walls of medium cuticularization; basal pharyngeal pores are large and conspicuous, but lack pharyngeal knobs; intestine broadest in front, narrowing gradually to the rear, with a bulge at the level of the ventral anus, U93.

*Reproductive tract*: Hermaphroditic, protandrous to simultaneous; paired testes extend rearward from just behind the PhJIn, their vasa deferentia recurving from the rear to the fore and exiting behind together at U31; small developing ova (61 × 13 and 41 × 8 µm) occur bilaterally in the mid-gut region; neither frontal nor caudal organs were seen.

##### Ecology:

 Sparse (less than 10% of samples) in frequency of occurrence, rare to scarce (fewer than 1 to 3–5% of a sample) in abundance; *sublittoral* in very fine to medium fine, medium-well sorted silicious or carbonate sand at 0.53 m depth, occasionally in fine to coarse, poorly sorted sand at 6 m depth near the bases of beachrock slabs.

##### Geographical distribution:

 **MED SEA:** *CYPRUS:* {Coral Bay [video]}; *EGYPT:* {^Bir Mesud (31°14'N, 29°58'E) [video], Cleopatra Beach [video], Green Beach, Hannoville, Mamura [4-videos]}; *ISRAEL:* {Palmachim N [video]}.

##### Remarks:

There are eight video sequences of *Paraturbanella levantia* sp. n., all from the eastern Mediterranean Sea in Cyprus, Egypt and Israel. Five of these are available as MPEG 2 (and MPEG 1) from Hummon (2009): #975 a mature **Lectotype** adult of Lt=657 µm (LPh=168 µm), collected in April 1994 from Bir Mesud, near Alexandria, Egypt; #976 a mature adult of Lt=635 µm (LPh=163 µm) from Coral Bay, Cyprus; #974 a subadult of Lt=400 µm (LPh=138 µm) from Cleopatra Beach, in Alexandria; #972 a subadult of Lt=335 µm (LPh=127 µm) from Mamura, near Alexandria; and the other, #971 a juvenile of Lt=211 µm (LPh=96 µm) also from Mamura. The Cleopatra specimen alone showed a caudal cone; others had an associated gland, but no protruding cone. Specimens often bear a variety of small diatoms internally. [*Note: some proposed species names were included in the prototype CD, referred to by Todaro et al. 2003, but were expurgated from the CD before it was made available to all attendees at the 11th International Meiofauna Conference of 2001 in Boston.]

##### Etymology:

 Levantia is named after the eastern Mediterranean region in which it was first found.

##### Taxonomic affinities:

 *Paraturbanella levantia* sp. n. is the only species in the genus to have a narrowly protruding outwardly rolled mouth, prominent pestle organs at U04, and a PhJIn at U26–U23, which also has TbA 8 per side, the medial tube shorter than the others; TbL absent; TbD 7 per side and TbV 14 per side, all in the intestinal region; TbP 8 per side, the outer being the longest and thickest; ‘dohrni’ [Seitenfüsschen] tubes 2 per side; but usually no caudal cone. *Paraturbanella levantia* sp. n., alone in the genus has both TbD and TbV, but lacks TbL.

#### Genus Turbanella Remane, 1925

##### Turbanella erythrothalassia sp. n.

urn:lsid:zoobank.org:act:9E249229-CDE5-4067-B0AF-4D702A17B586

http://species-id.net/wiki/Turbanella_erythrothalassia

[Trb erth]

Figure 18

Turbanella  EgyA Hummon (2009) [E Med & Red Seas Data Base].

###### Diagnosis:

 Adult Lt 440–486 µm; PhJIn at U33. Body medium-short, slender; head sculptured, with a narrow band of circumcephalic cilia and shallow lateral lobes at U05, but no tentacles; neck constriction greatest along the mid-pharynx; trunk broadest along the mid-body, thining gradually to the caudal base; caudum is slightly cleft, incised from its tips to U98, bearing a medial cone. Glands 36–37 per side, small, inconspicuous. TbA 7 per side, inner-most shorter than the others, on fleshy hands that insert at U11; TbVL 9 per side, with 1 along the fore half and 0 in the rear half of the pharynx, and the other 8 evenly spaced and symmetrically arranged along the intestine, and none behind the anal opening; ‘cirrata’ [Seitenfüsschen] tubes are short, but present at U35; TbP 7 per side, lengthening medial to lateral, inserting along the trailing edge of each lobe. Locomotor ciliature forms 2 longitudinal bands from buccal cavity to anus. Mouth terminal, of medium width; buccal cavity shallow, mugshaped; walls lightly cuticularized; pharynx broad, with conspicuous basal pharyngeal pores; intestine narrows fore to aft, with a slight bulge at the ventral anus, U92. Hermaphroditic; paired testes extend back from the PhJIn, its vasa deferentia recurving to the fore and probably exiting just behind the PhJIn, sperm sometimes descending in clusters to the base of the vasa deferentia; the bilateral ova occur in the fore-gut region and develop rear to fore; frontal and caudal organs not seen.

###### Description:

**Figure 18.**
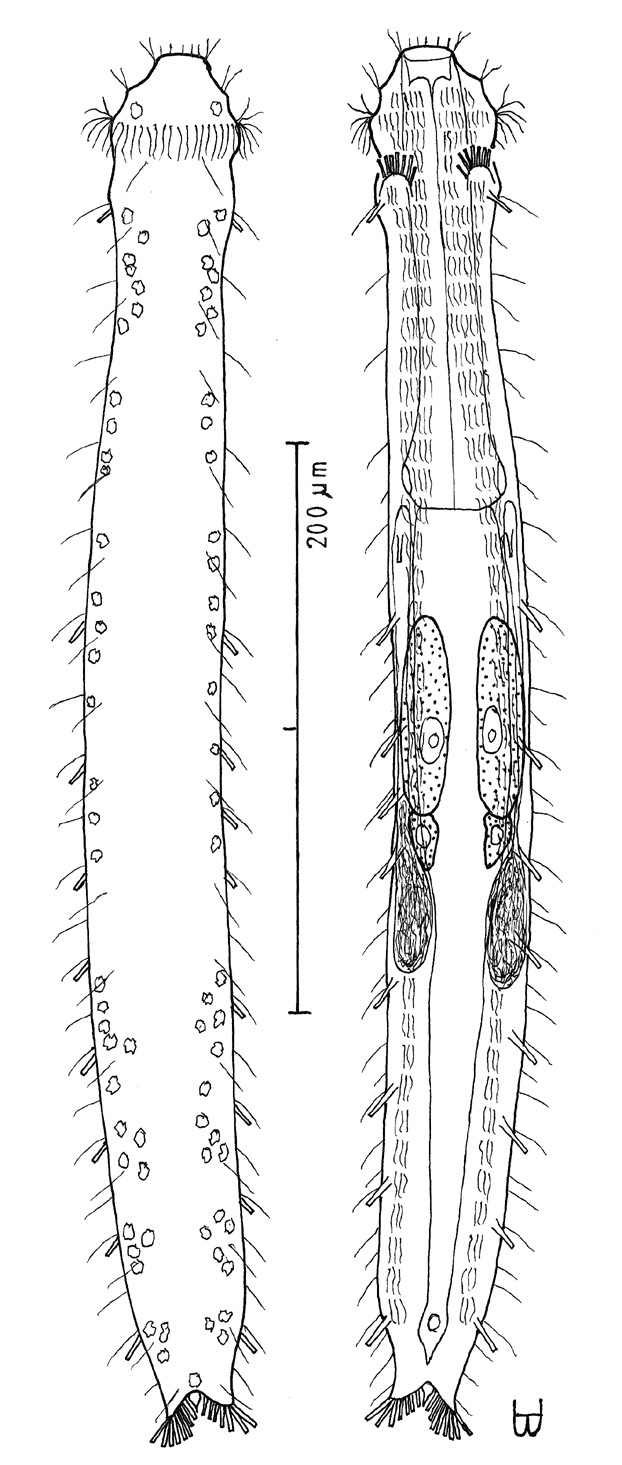
*Turbanella erythrothalassia* sp. n. dorsal and ventral views of a mature adult (Lt=486, LPh=166 µm) from Moon Valley, Hurghada, Egypt; dorsal with pattern of glands and dorsal and lateral body cilia; ventral with digestive and reproductive tracts, adhesive tubes and locomotor ciliary bands.

 Adult Lt 440–486 µm; L to PhJIn 162–166 µm at U38–U33 (Fig. 18). Body medium-short, slender; head sculpted, with a narrow band of circumcephalic cilia and shallow lateral lobes at U05, but no tentacles; neck constriction greatest along the mid-pharynx; trunk broadest along the mid-body, thinning gradually to the caudal base; caudum is slightly cleft, incised from its tips to U98, bearing a medial cone. Widths of apex /head at turban /trunk at PhJIn /mid-gut /furcal base, tips, and their locations along the body length are: 46 /37 /47 /49 /24, 39 µm at U06 /U16 /U33 /U52 /U96, U100. Glands 36–37 per side, small (diameter 3–4 µm), scattered and inconspicuous.

*Adhesive tubes*: TbA 7 per side, inner-most shorter than the others, occuring on fleshy hands that insert at U11; TbVL 9 per side (L 10–14 µm), with 1 along the fore half at U11 and 0 along the rear half of the pharynx, and 8 evenly spaced and symmetrically arranged along along the intestine at U40–U91, none behind the anal opening; ‘cirrata’ [Seitenfüsschen] tubes are present at U35 but short (L 9 µm); TbP 7 per side, lengthening from medial to lateral (L 6–10 µm), arrayed along the rear edge of each lobe.

*Ciliature*: Mouth is surrounded with short sensory cilia (L 4 µm), with 2 pairs of longer vibratile cilia (L 8–10 µm) per side inserted at the points of head sculpting; ciliary hairs (L 12 µm) form a circumcephalic band at U05; sensory cilia of similar length (L 14–16 µm) occur on the trunk in lateral and dorsal columns, with 20 and 16 per side, respectively. Ventral locomotor cilia (L 8 µm) run rearward in 2 longitudinal bands that trace the lateral body margins, remaining separate throughout, the cilia occurring in transverse rows.

*Digestive tract*: Mouth terminal, of medium width (18 µm diameter); buccal cavity mugshaped, shallow; walls lightly cuticularized; pharynx broad throughout, with conspicuous basal pharyngeal pores; intestine broadest in the fore-gut, narrowing gradually toward the rear, with a slight bulge at the ventral anus at U92.

*Reproductive tract*: Hermaphroditic; paired testes extend rearward from the PhJIn, sperm sometimes descending in clusters to the base of the vasa deferentia, male seminal pore not seen; bilateral ovules and ova (to 71 × 18 µm) occur in the fore-gut region and develop rear to fore; neither frontal, nor caudal organs were seen.

###### Ecology:

 Sparse in frequency of occurrence (fewer than 10% of samples), scarce to prevalent in abundance (less than 3–5% to greater than 30% of a sample, sometimes a dominant **[dom]**); *littoral* in very fine to medium, medium sorted, clean silicious or corraline sand at low water neap to low water spring, 0–15 cm depth (occasionally in very fine to coarse, poorly sorted sand at mean tide level, 030 cm depth).

###### Geographical distribution:

 **RED SEA:** *EGYPT:* {Sharm el-Arab Inside, ^Moon Valley [video], Na’ama Bay N **[dom]**, Na’ama Bay S [2-videos], Sharm el-Naga, Ras Sudr **[dom]**, ’Uyun Musa}; *ISRAEL:* {N Beach [2-videos]}.

###### Remarks:

There are five video sequences of *Turbanella erythrothalassia* sp. n., all from Egypt and Israel in the upper Red Sea. Three of these are available as MPEG 2 (and MPEG 1) from Hummon (2009): #1058 a mature **Lectotype** adult of Lt=486 µm (LPh=153 µm), collected in June 1994 from Moon Valley, Hurghada, Egypt; #1054 a mature adult of 440 µm (LPh=166 µm) from North Beach, Eilat, Israel; and #1059 a subadult of Lt=413 µm (LPh=145 µm) from Na’ama Bay S. Sinai, Egypt. *Turbanella erythrothalassia* sp. n. is unusual among members of the genus in having ventral locomotor cilia arranged in transverse lines.

###### Etymology:

 Erythrothalassia (Greek: *erythros* + *thalassa* = meaning ‘red sea’) referring to the Red Sea in which it was found.

###### Taxonomic affinities:

 *Turbanella erythrothalassia* sp. n. is the only species in the genus with a sculptured head, having shallow lobes, a PhJIn at U38–U33, a medium mouth, and a body with a mid-pharyngeal neck, which also has TbA 7 per side the inner-most being the shortest; a TbVL formula of 9=1,0/8,0 (1 along the fore half and 0 along the rear half of the pharynx, 8 along the intestine and none behind the anus); with short ‘cirrata’ [Seitenfüsschen] tubes at U35; and TbP 7 per side, along the rear edge of each caudal lobe; and a caudal cone, but without TbV or TbD. The species nearest *Turbanella erythrothalassia* sp. n. is *Turbanella pacifica* Schmidt, 1974, which though somewhat smaller, has a similar body conformation and also lacks TbD; however, they differ in the numbers of adhesive tubes (TbA: 7 in the former, 4–5 in the latter; TbL 9 in the former, with none along the rear pharynx, 12 in the latter, with 1 along the rear pharynx; TbP 7 in the former, with a caudal cone, 6 in the latter, without a caudal cone; ‘cirrata’ tube present in the former, absent in the latter).

## Supplementary Material

XML Treatment for Cephalodasys dolichosomus

XML Treatment for Cephalodasys saegailus

XML Treatment for Dactylopodola agadasys

XML Treatment for Dendrodasys rubomarinus

XML Treatment for Macrodasys imbricatus

XML Treatment for Macrodasys macrurus

XML Treatment for Macrodasys nigrocellus

XML Treatment for Macrodasys scleracrus

XML Treatment for Urodasys toxostylis

XML Treatment for Tetranchyroderma corallium

XML Treatment for Tetranchyroderma rhopalotum

XML Treatment for Tetranchyroderma sinaiensis

XML Treatment for Tetranchyroderma xenodactylum

XML Treatment for Paraturbanella levantia

XML Treatment for Turbanella erythrothalassia
